# Angiotensin-converting enzyme 2 identifies immuno-hot tumors suggesting angiotensin-(1–7) as a sensitizer for chemotherapy and immunotherapy in breast cancer

**DOI:** 10.1186/s12575-022-00177-9

**Published:** 2022-10-25

**Authors:** Jie Mei, Yun Cai, Rui Xu, Xinqian Yu, Xu Han, Miaomiao Weng, Lingyan Chen, Tao Ma, Tianshu Gao, Fei Gao, Tiansong Xia, Yichao Zhu, Yan Zhang

**Affiliations:** 1 Department of Oncology, Wuxi Maternal and Child Health Hospital Affiliated to Nanjing Medical University, No. 48 Huaishu Rd, Wuxi, 214000 China; 2 Wuxi Clinical Medical College, Nanjing Medical University, Wuxi, 214000 China; 3 The First Clinical Medical College, Nanjing Medical University, Nanjing, 211166 China; 4 Department of Physiology, Nanjing Medical University, No. 101 Longmian Av, Nanjing, 211166 China; 5 Jiangsu Breast Disease Center, the First Affiliated Hospital With Nanjing Medical University, No. 300 Guangzhou Rd, Nanjing, 210029 China; 6 Department of Breast Surgery, Wuxi Maternal and Child Health Hospital Affiliated to Nanjing Medical University, Wuxi, 214000 China

**Keywords:** ACE2, Breast cancer, Tumor microenvironment, Ang-1–7, Immunotherapy

## Abstract

**Background:**

Angiotensin-converting enzyme 2 (ACE2) is known as a tumor suppressor and lowly expressed in most cancers. The expression pattern and role of ACE2 in breast cancer (BC) have not been deeply elucidated.

**Methods:**

A systematic pan-cancer analysis was conducted to assess the expression pattern and immunological role of ACE2 based on RNA-sequencing (RNA-seq) data downloaded from The Cancer Genome Atlas (TCGA). The correlation of ACE2 expression and immunological characteristics in the BC tumor microenvironment (TME) was evaluated. The role of ACE2 in predicting the response to therapeutic options was estimated. Moreover, the pharmacodynamic effect of angiotensin-(1–7) (Ang-1–7), the product of ACE2, on chemotherapy and immunotherapy was evaluated on the BALB/c mouse BC model. In addition, the plasma samples from BC patients receiving neoadjuvant chemotherapy were collected and subjected to the correlation analysis of the expression level of Ang-1–7 and the response to neoadjuvant chemotherapy.

**Results:**

ACE2 was lowly expressed in BC tissues compared with that in adjacent tissues. Interestingly, ACE2 was shown the highest correlation with immunomodulators, tumor-infiltrating immune cells (TIICs), cancer immunity cycles, immune checkpoints, and tumor mutation burden (TMB) in BC. In addition, a high level of ACE2 indicated a low response to endocrine therapy and a high response to chemotherapy, anti-ERBB therapy, antiangiogenic therapy and immunotherapy. In the mouse model, Ang-1–7 sensitized mouse BC to the chemotherapy and anti-PD-1 immunotherapy, which revealed its significant anti-tumor effect. Moreover, a high plasma level of Ang-1–7 was associated with a better response to neoadjuvant chemotherapy.

**Conclusions:**

ACE2 identifies immuno-hot tumors in BC, and its enzymatic product Ang-1–7 sensitizes BC to the chemotherapy and immunotherapy by remodeling the TME.

**Supplementary Information:**

The online version contains supplementary material available at 10.1186/s12575-022-00177-9.

## Background

It has been well-knowlegded that ACE2 is tissue-specifically distributed and preferentially expressed in the lung, esophagus, kidney, bladder, testis, stomach and ileum [1]. ACE2 is known as a tumor suppressor and lowly expressed in most cancers [2–4]. Recently, study presented by Khanna et al*.* reports that ACE2 abrogates tumor resistance to VEGFR inhibitors and its enzymatic product angiotensin-(1–7) (Ang-1–7) is a potential therapeutic drug for clear cell renal cell carcinoma [5]. All these evidences suggested that ACE2 might play a critical role in the initiation and progression of human cancers.

Breast cancer (BC) is a multifactorial disease, which has the highest incidence in the world. In 2020, a total of 2,261,419 new cases and 684,996 deaths have been reported [6]. The downregulation of ACE2 promotes metastasis of BC via Ang-1–7 [7]. Encouragingly, several analyses reveal that ACE2 correlates with the abundance of a number of tumor-infiltrating immune cells (TIICs) in multiple cancers [8, 9]. However, indiscriminate pan-cancer analysis neglects in-depth research on dominant tumor species, which may lead to the ignoring of the great value of ACE2 in regulating tumor immunity.

Tumors are complex masses consisting of malignant cells as well as normal cells. The intercellular multiple components, such as cytokines, chemokines, growth factors, etc., constitute the tumor microenvironment (TME) [10]. Tumors can be simply classified into “cold” or “hot” depending on the features of TME. “Cold” tumors are characterized as immunosuppressive TME and insensitive to either chemotherapy or immunotherapy, while “hot” tumors represent high response rates to these therapies, which is featured by T cell infiltration and immunoactivated TME [11]. In principle, the hot tumors exhibit a better response to immunotherapy, such as anti-PD-1/PD-L1 therapy [12]. Thus, the stratification of hot and cold tumors is a critical strategy to demarcate the response to immunotherapy.

In this research, a pan-cancer analysis of the expression and immunological features of ACE2 revealed its tight correlation with immunological factors in BC. The high expression of ACE2 indicated an inflamed TME and identified immuno-hot tumors in BC, and had the potential to estimate the molecular subtype of BC. We also reported that Ang-1–7 could be a novel predictive biomarker for neoadjuvant chemotherapy and a sensitizer for chemotherapy and immunotherapy.

## Methods and materials

### Public datasets retrieval

The Cancer Genome Atlas (TCGA) data: The pan-cancer normalized RNA-seq datasets, copy number variant (CNV) data processed by GISTIC algorithm, 450 K methylation data, mutation profiles, and clinical information were obtained from UCSC Xena data portal (https://xenabrowser.net/datapages/). The somatic mutation data were obtained from the TCGA (http://cancergenome.nih.gov/) database and then used to calculate the tumor mutation burden (TMB) by R package “maftools”. The abbreviations for TCGA cancer types were shown in Table S1.

The METABRIC data: The normalized RNA-seq dataset, CNV data processed by GISTIC algorithm, mutation profiles, and clinical data in the METABRIC cohort were downloaded from the cBioPortal data portal (http://www.cbioportal.org/datasets) [13]. The sample size for each dataset was shown in Table S2.

### Pan-cancer analysis of the correlation between ACE2 and immunological features

To evaluate the pan-cancer immunological correlation of ACE2, we first collected expression levels of 122 immunomodulators including major histocompatibility complex (MHC), receptors, chemokines, and immunostimulators from the study of Charoentong et al*. * [14]. Then, the correlations between ACE2 and immune checkpoints were also assessed. The TISIDB [15] tool was used to estimate the abundance of immune cells infiltration, and the correlations between ACE2 and the infiltration levels of immune cells were next evaluated.

### Linked Omics database analysis

The Linked Omics database (http://www.linkedomics.org/login.php) is a web-based tool to analyze multi-dimensional datasets [16]. The functional roles of ACE2 in BC were predicted using the Linked Omics tool in term of Gene Ontology (GO) and Kyoto Encyclopedia of Genes and Genomes (KEGG) pathways by the gene set enrichment analysis (GSEA). Default options were used for all parameters.

### Assessment of the immunological features in TME of BC

The immunological features of TME in BC contained immunomodulators, the activities of the cancer immunity cycle, infiltration levels of TIICs, and the expression of inhibitory immune checkpoints.

The expressions of 122 immunomodulators were first included in this part. Considering the cancer immunity cycle which contains seven stages reflects the anti-cancer immune response and the activities of each step decide the fate of tumor cells, we subsequently calculated the activation scores of each step by the single-sample gene set enrichment analysis (ssGSEA) according to the expression level of specific signatures of each step [17]. The ssGSEA algorithm, implemented by extending GSEA method, allows the definition of an enrichment score that represents the absolute enrichment of gene sets in each sample within a given dataset. The gene expression profile of the given dataset was sorted and normalized firstly. Then, the enrichment scores were assessed based on the empirical cumulative distribution function of signatures in the gene sets and remaining genes. Given of the biological and technical batch effects among datasets came from different researches and the algorithm schedule, ssGSEA algorithm was applied to calculate the enrichment scores of samples for each dataset respectively, rather than for samples in the integrated dataset. Meanwhile, all bioinformatic analysis was performed for each dataset, respectively. Moreover, in order to avoid calculation errors resulting from various algorithms which were developed to explore the relative abundance of TIICs in TME, we comprehensively estimated the infiltration levels of TIICs using five independent algorithms: TIMER [18], EPIC [19], MCP-counter [20], quanTIseq [21] and TISIDB [15]. The ESTIMATE algorithm was also performed to calculate Tumor Purity, ESTIMATE Score, Immune Score and Stromal Score [22]. Furthermore, we also collected several well-known effector genes of TIICs, and computed the T cell inflamed score according to the linear combination of the expression levels and weighting coefficient of 18 genes reported by Ayer et al*. * [23].

To verify the role of ACE2 in mediating cancer immunity in BC, we grouped the patients into the high ACE2 and the low ACE2 group with the 50% cutoff based on the median expression levels of ACE2, and then analyzed the difference of the immunological features of TME concerning the above aspects between the high and the low ACE2 groups.

### Immunophenoscore analysis

As previously reported, a patient’s immunophenoscore (IPS) can be calculated without bias using machine learning by consideration of the 4 major categories of components that measure immunogenicity: effector cells, immunosuppressive cells, MHC molecules, and immunomodulators [14]. The IPS values of BC patients were obtained from the Cancer Immunome Atlas (TCIA) (https://tcia.at/home).

### Calculation of the enrichment scores of various gene signatures

According to previous research [24], we collected several gene-sets positively associated with therapeutic responses to immunotherapy, targeted therapy and radiotherapy and specific markers of biological processes correlated with anti-tumor immunity. The enrichment scores of these signatures were obtained using the “GSVA” R package [25]. Detailed information on immunotherapy-related gene signatures was shown in Table S3.

### Prediction of therapeutic response

The role of ACE2 in predicting the response to chemotherapy was also evaluated. First, BC-related drug-target genes were screened by using the Drugbank database (https://go.drugbank.com/). The R package “pRRophetic” where the samples’ half-maximal inhibitory concentration (IC50) was calculated by ridge regression were utilized to predict the response to anti-cancer therapy for patients in different cohots. The statistical models were firstly built based on the gene expression and drug sensitivity data in a considerably large panel of cancer cell lines obtained from the Cancer Genome Project (CGP) database (https://www.sciencedirect.com/topics/neuroscience/cancer-genome-project). Then, these models were combinated the gene expression profiles from tumor biopsies of different cohorts to predict the clinical drug response, respectively. Meanwhile, the tenfold cross validation on the test set was performed to estimate the accuracy of the phenotype prediction.Default options were used for all parameters [26].

### Clinical samples

Two tissue microarrays (TMAs, HBreD050Bc01 and HBreD090Bc03) were obtained from Outdo Biotech (Shanghai, China). The TMAs were embedded in paraffin, and the thickness of the TMAs was 4 μm. The HBreD050Bc01 microarray contained 40 BC and 10 adjacent samples. The HBreD090Bc03 microarray contained 85 BC and 5 adjacent samples. Thus, a total of 125 BC samples and 15 adjacent samples were involved in the current research. Ethical approval (YBM-0502, 2020–03) for this part was granted by the Clinical Research Ethics Committee, Outdo Biotech.

In addition, 30 plasma samples from BC patients receiving the Docetaxel-based neoadjuvant chemotherapy were obtained before and after they received chemotherapy. The detailed information for these patients was shown in Table S4. These BC patients were recruited by the First Affiliated Hospital with Nanjing Medical University. After 8 cycles of neoadjuvant chemotherapy, they received a surgical operation. The response to neoadjuvant chemotherapy was evaluated according to the Miller-Payne criterion. Ethical approval (NJMU-2020–93, 2020–03) for this part was granted by the Clinical Research Ethics Committee, Nanjing Medical University.

### Immunohistochemistry and semi-quantitative evaluation

Next, Immunohistochemistry (IHC) staining was conducted on these tissue slides. The primary antibodies used in the research were as follows: anti-ACE2 (1:3000 dilution, Cat. ab15348, Abcam, Cambridge, UK), anti-CD8 (Ready-to-use, Cat. PA067, Abcarta, Suzhou, China), and anti-PD-L1 (Ready-to-use, Cat. GT2280, GeneTech, Shanghai, China). Antibody staining was visualized with DAB and hematoxylin counterstain, and stained sections were scanned using Aperio Digital Pathology Slide Scanner. All stained sections were independently evaluated by two independent pathologists. For semi-quantitative evaluation of ACE2 and PD-L1 staining, the immunoreactivity score (IRS) was applied as previously described [27]. For CD8 staining, infiltration level was assessed by estimating the percentage of cells with strong intensity of membrane staining in the stroma cells. In addition, tumors were demarcated into three phenotypes based on the spatial distribution of CD8^+^ T cells, including the inflamed, the excluded, and the deserted phenotypes [28].

### Enzyme-linked immunosorbent assay (ELISA)

The levels of plasma Ang-1–7 in BC patients were detected by the ELISA assay. The ELISA kit for Ang-1–7 (Cat. E-EL-H5518) was obtained from Elabscience (Wuhan, China). All samples were assayed in duplicates according to the manufacturer’s protocol, and the average values were reported as pg/mL. The association between plasma Ang-1–7 levels and the response to neoadjuvant chemotherapy was next assessed.

### Cell culture and plasmid transfection

MDA-MB-231 (RRID: CVCL_0062) and 4T1 (RRID: CVCL_0125) cell lines were purchased from KeyGEN BioTECH (Nanjing, China). MDA-MB-231 cells were maintained in Leibovitz’s L-15 medium (KeyGEN BioTECH, Nanjing, China) supplemented with 10% fetal bovine serum (FBS) (Hyclone, Thermo Scientific, Waltham, MA) at 37 °C with 5% CO_2_. 4T1 cells were maintained in DMEM medium (KeyGEN BioTECH, Nanjing, China) supplemented with 10% FBS at 37 °C with 5% CO_2_. All experiments were performed with mycoplasma-free cells. MDA-MB-231 cell lines have been authenticated using short tandem repeat profiling. The ACE2 overexpression plasmid were synthesized by KeyGEN BioTECH (Nanjing, China). The cloning vector for ACE2 overexpression plasmid was pcDNA3.1( +)-EGFP. For subsequent assays, MDA-MB-231 cells were transfected with ACE2 plasmid (2.5 μg) using Lipofectamine 3000 Reagent (Invitrogen) according to the manufacturer’s instructions.

### Quantitative real‑time PCR (qRT-PCR)

Total RNA of BC cells was extracted using TRIzol reagent (Invitrogen). The primers for *ACE2*, *PD-L1* and *GAPDH* mRNA reverse transcription were synthesized in KeyGEN BioTECH (Nanjing, China). qRT-PCR was conducted using the One Step TB Green™ PrimeScript™ RT-PCR Kit II (SYBR Green, TaKaRa). Primers used for gene amplification as following: ACE2: 5’ GCTCTTCCTGGCTCCTTCTCAG 3’ (forward), 5’ AGGTCTTCGGCTTCGTGGTTAA 3’ (reverse); PD-L1: 5’ GCCGAAGTCATCTGGACAAGC 3’ (forward), 5’ TGATTCTCAGTGTGCTGGTCAC 3’ (reverse); GAPDH: 5’ AGATCATCAGCAATGCCTCCT 3’ (forward), 5’ TGAGTCCTTCCACGATACCAA 3’ (reverse). The 2^−ΔΔCt^ method was used for mRNA expression analysis.

### Western blotting analysis

Cells were plated in 35-mm dishes (6 × 10^5^ cells/dish). 48 h after transfection, all cells were harvested the proteins with lysis buffer. SDS–polyacrylamide gel electrophoresis and Western blotting analysis were performed as standard protocols. For nuclear and cytoplasmic protein extraction, nuclear and cytoplasmic Protein Extraction Kit were used (KeyGEN BioTECH, Nanjing, China). The primary antibodies for ACE2 (1:1000 dilution, Cat. ab15348, Abcam), PD-L1 (1:1000 dilution, Cat. 17,952–1-AP, ProteinTech) and Tubulin (1:2000 dilution, Cat. 10,094–1-AP, ProteinTech) were used. Expression levels of proteins were normalized to Tubulin for each sample.

### Cell Counting Kit-8 (CCK-8) assay

In general, BC cells were grouped into three groups: blank control group, negative control group (transfected with control plasmid) and ACE2 overexpression group. Twenty-four hours after transfection, cells were digested using 0.25% trypsin for 1 min and resuspended. Suspended cells were seeded on a 96-well plate with the cell density adjusted to 2 × 10^4^ cells/ml (100 μl/well) and fostered at 37 °C in a constant-temperature incubator with 5% CO_2_ for 24, 48, 72 and 96 h, respectively. To each well, 10 μl CCK-8 was added, after which the plate was put in the incubator for 2 h. The OD value of each well was measured at 450 nm by a microplate reader. Each experiment was repeated three times.

### Mouse BC model and pharmacodynamic evaluation

For subcutaneous tumor models, female BALB/c mice purchased from Shanghai SLAC Laboratory Animal Co.,Ltd were used in in vivo analysis. The mice were housed and maintained in laminar flow cabinets under specific pathogen-free conditions. All experiments were approved by the Laboratory Animal Ethics Committee at Nanjing Medical University (IACUC-1902016, 2019–02). To establish BC tumor xenografts, 4T1 mouse tumor cells were subcutaneously injected (1 × 10^7^ cells) into the flanks of 5- to 6-week-old female mice (~ 20 g on average). Tumors were monitored and regularly measured with calipers every two to three days. When tumors reached about 100 mm^3^ in volume, mice were randomized into six different groups: vehicle group, Ang-1–7 group, Docetaxel group, Ang-1–7 + Docetaxel group, anti-PD-1 and Ang-1–7 + anti-PD-1 group. Ang-1–7 (Cat. HY-12403, MedChemExpress) and Docetaxel (Cat. D107320, Aladdin) were dissolved in PBS. Ang-1–7 was administered to mice through subcutaneous injection at 1 mg/kg daily. Docetaxel was administered to mice through intraperitoneal injection at 10 mg/kg three times a week. Anti-PD-1 antibody (BioXCell, Cat. BE0273) was dissolved in PBS, and 200 μg was administered intraperitoneally three times a week. The vehicle solution consisted of PBS only. Treatment was continued until tumors reached 20 mm along the long axis or until 25 days after treatment initiation.

To evaluate the infiltrating levels of immune cells in control and Ang-1–7 treated tumors, IHC staining was used. Tumors were fixed in 4% paraformaldehyde for 20 min and permeabilized with 0.5% Triton X-100 in PBS for 10 min. Tumor sections were blocked with BSA 5% for 30 min. The sections were incubated with primary antibodies against CD8 (1:100 dilution, Cat. A11856, ABclonal, Wuhan, China) and CD3 (1:1000 dilution, Cat. 17,617–1-AP, ProteinTech, Wuhan, China) at 4 °C overnight and the corresponding secondary antibody. After washing, DAPI was used for the nuclei staining. Images were scanned using OlyVIA Slide Scanner.

To detect the expression of effective and suppressive cytokines in control and Ang-1–7 treated tumors, ELISA test was used. The ELISA kit for IFN-γ (Cat. E-EL-M0048), CXCL9 (Cat. E-EL-M0020), CXCL10 (Cat. E-EL-M0021), TGF-β1 (Cat. E-EL-0162), IL-10 (Cat. E-EL-M0046) were obtained from Elabscience (Wuhan, China). The values were standardized by protein concentration of each sample and were reported as pg/mg or ng/mg.

## Statistical analysis

Statistical analysis and figure exhibition were performed using R language 4.0.0. The difference between the two groups was analyzed by parametric Student’s t-test or non-parametric Mann Whitney test, and the difference between multiple groups was analyzed by parametric one-way ANOVA followed by Tukey’s multiple comparisons test. Pearson correlation analysis was used to evaluate the correlation between two variables. For all analyses, *P* value < 0.05 was deemed to be statistically significant and labled with *P < 0.05, **P < 0.01, ***P < 0.001, ****P < 0.0001.

## Results

### Expression and immunological roles of ACE2 across human cancers

After a systematic pan-cancer analysis of the expression of ACE2 in the TCGA database, we discovered that ACE2 was lowly expressed in a fraction of cancers, including BRCA, KICH, and LUAD. However, ACE2 was overexpressed in CESC, KIRP, LIHC and UCEC (Figure S1). Next, a pan-cancer analysis was conducted to examine the immunological correlations of ACE2 in various tumors. The results uncovered that ACE2 was positively correlated with most immunomodulators in BC (Figure S2A). We also calculated the infiltrating levels of TIICs in the TME by the ssGSEA method. Similarly, ACE2 was positively correlated with most TIICs in BC (Figure S2B). Moreover, we revealed that the expression of ACE2 was positively related to the expression of several immune checkpoints, including LAG3, TIGIT, CTLA4, and PD-L1 in BC (Figure S2C). Although these positive correlations between ACE2 and immunological features were found in other tumors, such as CESC, KIRC and PRAD, the highest correlation was emerged in BC. According to a previous research, ACE2 was not expressed in immune cells, and the expression of ACE2 in bulk RNA-seq data was derived from non-immune cells in all probability, such as tumor cells in the tissues [29]. We also visualized the expression patterns of ACE2 in different cell types in BC tissues using the TISCH tool (http://tisch.comp-genomics.org/home/). The result showed that ACE2 highly expressed in tumor cells (Figure S3A), which was also validated by the IHC statining (Figure S3B-C). Collectively, the expression pattern of ACE2 is TME-characteristic, which indicates the potential of ACE2 as an immune-related biomarker and therapeutic target in BC.

### Potential regulatory factors and functions of ACE2 in BC

Mutations in ACE2 gene were rare (Figure S4A), so the mutations seemed not to be a dominat factor for ACE2 expression. The CNV pattern of ACE2 was shown in Figure S4B. Remarkably, the amplification of ACE2 upregulated the expression of *ACE2* mRNA (Figure S4B). In addition, the methylation level was positively correlated with *ACE2* mRNA expression (Figure S4C). These findings suggest that epigenetic modifications of the *ACE2* may be essential for the regulation of ACE2 expression.

Moreover, the functions of ACE2 in BC were analyzed using the LinkedOmics tool. GO enrichment analysis assessed the functions of ACE2 in terms of three aspects, including biological processes, cellular components and molecular functions. Plentiful statistically significant terms were found and the top 5 terms positively correlated with ACE2 expression of each analysis were retained. As shown in Table S5, the most critical terms were associated with immune-related processes. These results reveal that ACE2 may act as a critical role in regulating anti-tumor immunity in BC.

### ACE2 is associated with an inflamed TME in BC

**Fig. 1:**
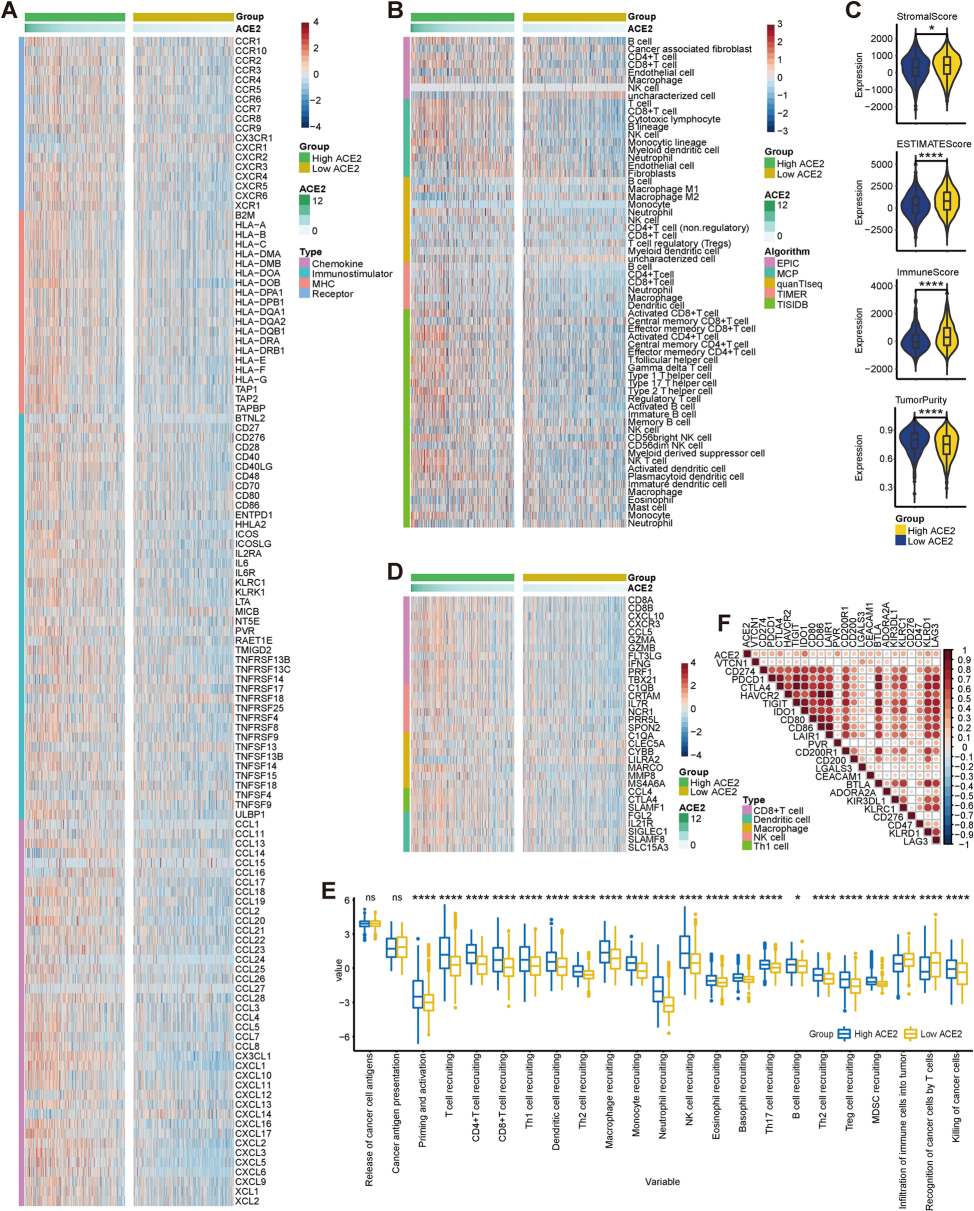
ACE2 predicts an inflamed TME in BC. **A** Expression levels of 122 immunomodulators between the high and low ACE2 groups in BC. **B** Differences in the levels of TIICs calculated using five algorithms between the high and low ACE2 groups. **C** Differences in Tumor Purity, ESTIMATE Score, Immune Score, and Stromal Score estimated by the ESTIMATE method between the high and low ACE2 groups. Significance was calculated with Student’s t-test. *P < 0.05, ****P < 0.0001. **D** Differences in the gene markers of the common TIICs between the high and low ACE2 groups. **E** Differences in the various steps of the cancer immunity cycle between the high and low ACE2 groups. Significance was calculated with Student’s t-test. ns-*P* > 0.05, *P < 0.05, ****P < 0.0001. **F** Correlation between ACE2 and common inhibitory immune checkpoints. Significance was calculated with Pearson correlation analysis. The color reveals the Pearson correlation coefficient

Considering that ACE2 was positively related to a great proportion of immunomodulators in BC, we next explored the in-depth immunological role of ACE2 in BC. Most MHC molecules were upregulated in the high ACE2 group, which suggested that the abilities of antigen presentation and processing were upregulated in the high ACE2 group (Fig. 1A). Most chemokines and paired receptors were upregulated in the high ACE2 group (Fig. 1A). These chemokines and receptors facilitate the recruitment of effector TIICs, including CD8^+^ T cells, TH17 cells and antigen-presenting cells. Next, we calculated the infiltration level of TIICs using five independent algorithms. Similar to the previous results, ACE2 was positively related to the infiltration levels of the majority of immune cells calculated using various algorithms (Fig. 1B). The ESTIMATE method was applied to estimate Tumor Purity, ESTIMATE Score, Immune Score, and Stromal Score. Compared with the low ACE2 group, the high ACE2 group had enhanced ESTIMATE Score, Immune Score, and Stromal Score, but decreased Tumor Purity (Fig. 1C). In addition, ACE2 was negatively correlated with the marker genes of immune cells, including CD8^+^ T cell, dendritic cell, macrophage, NK cell and Th1 cell (Fig. 1D). In addition, the activities of the cancer immunity cycle are a directly integrated manifestation of the functions of the chemokines and other immunomodulators. In the high ACE2 group, the activities of the most steps in the cycle were upregulated (Fig. 1E). The expression of inhibitory immune checkpoints, such as PD-L1, was adaptably upregulated in the inflamed TME [30]. ACE2 was suggested to be positively related to most immune checkpoints, including VTCN1, PD-L1, PD-1, CTLA4, etc. (Fig. 1F). Totally, ACE2 is tightly correlated with the development of an inflamed TME, which may act as a critical role in identifying the immunogenicity in BC.

### ACE2 predicts immune phenotype in BC

**Fig. 2:**
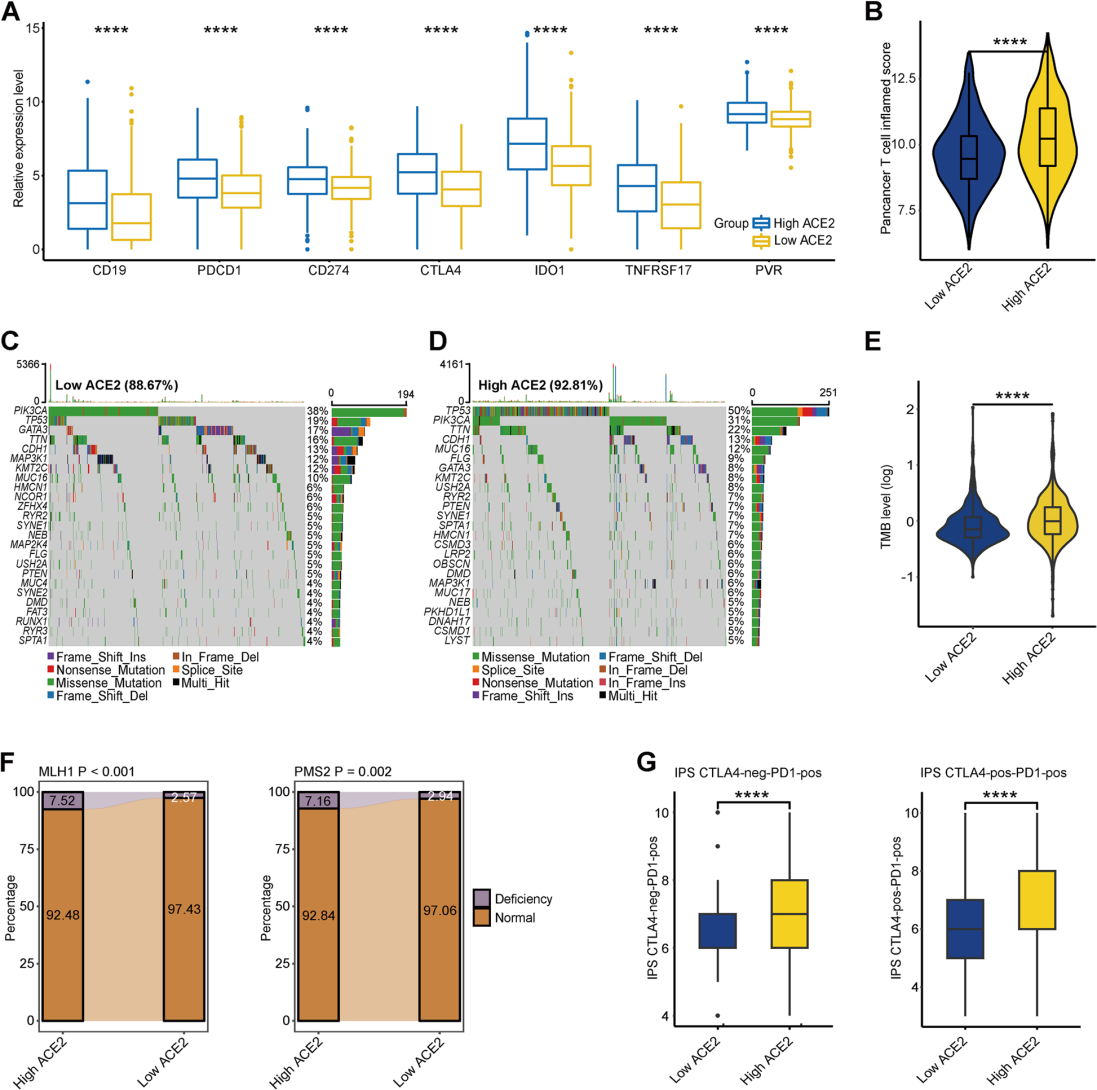
Correlation between ACE2 and the immune phenotype in BC. **A** Differences in expression levels of immune-related targets the high and low ACE2 groups in BC. Significance was calculated with Student’s t-test. ****P < 0.0001. **B** Differences in T cell inflamed scores between the high and low ACE2 groups. The T cell inflamed score is positively related to the response to cancer immunotherapy. Significance was calculated with Student’s t-test. ****P < 0.0001. **C**, **D** Mutational landscape in the high and low ACE2 groups. **E** Differences in TMB levels between the high and low ACE2 groups. Significance was calculated with Student’s t-test. ****P < 0.0001. **F** Differences in deficiency rates of MMR proteins between the high and low ACE2 groups. Significance was calculated with Pearson’s Chi-squared test. **G** Differences in IPS scores between the high and low ACE2 groups. Significance was calculated with Student’s t-test. ****P < 0.0001

Theoretically, BC patients with high ACE2 expression have high responses to immunotherapy. The expression levels of immune-related target proteins commonly reflect the response to immunotherapy. As expected, the expression levels of most immunotargets, such as CD19, PD-1 and PD-L1, were upregulated in the high ACE2 group (Fig. 2A). T cell inflamed score is developed using IFN-γ-related mRNA profiles to predict clinical response to PD-1 blockade [23]. We found that BC patients in the high ACE2 group exhibited higher T cell inflamed scores (Fig. 2B). TMB level is another biomarker for the prediction of the response to immunotherapy [31]. The frequency of mutant genes and TMB in the low ACE2 group were both lower compared with that in the high ACE2 group (Fig. 2C-E). Given that the TMB levels were mostly enriched in the range of 0–1200 (Figure S5), the comparison between the two groups was limited to the range of 1–1200 to avoid the effect of extremum. More importantly, TP53 exhibited incredibly high mutation rates in the high ACE2 group (Fig. 2C-D), which was a biomarker for good response to immunotherapy [32, 33]. According to previous research, patients with high levels of microsatellite instability (MSI-H) tend to be sensitive to immunotherapy [31]. We next assessed the status of mismatch repair (MMR) proteins and ACE2 expression. The proportion of MSI-H in BC was largely varied from 0.2% to 18.6% [34], but in numerous proportion of MSI-H in BC was less than 5%. We set the low 5% as the threshold of MMR protein deficiency. As expected, the proportion of MLH1 and PMS2 deficiency in the high ACE2 group was higher than that in the low ACE2 group, which indicated that BC patients with high ACE2 expression might have a high MSI-H proportion (Fig. 2F). In additon, we discovered that patients with high ACE2 expression had notably high IPS (Fig. 2G). In summary, immunotherapy may be carried out in BC patients with high ACE2 expression, who may be sensitive to immunotherapy.

### ACE2 predicts therapeutic options in BC

**Fig. 3:**
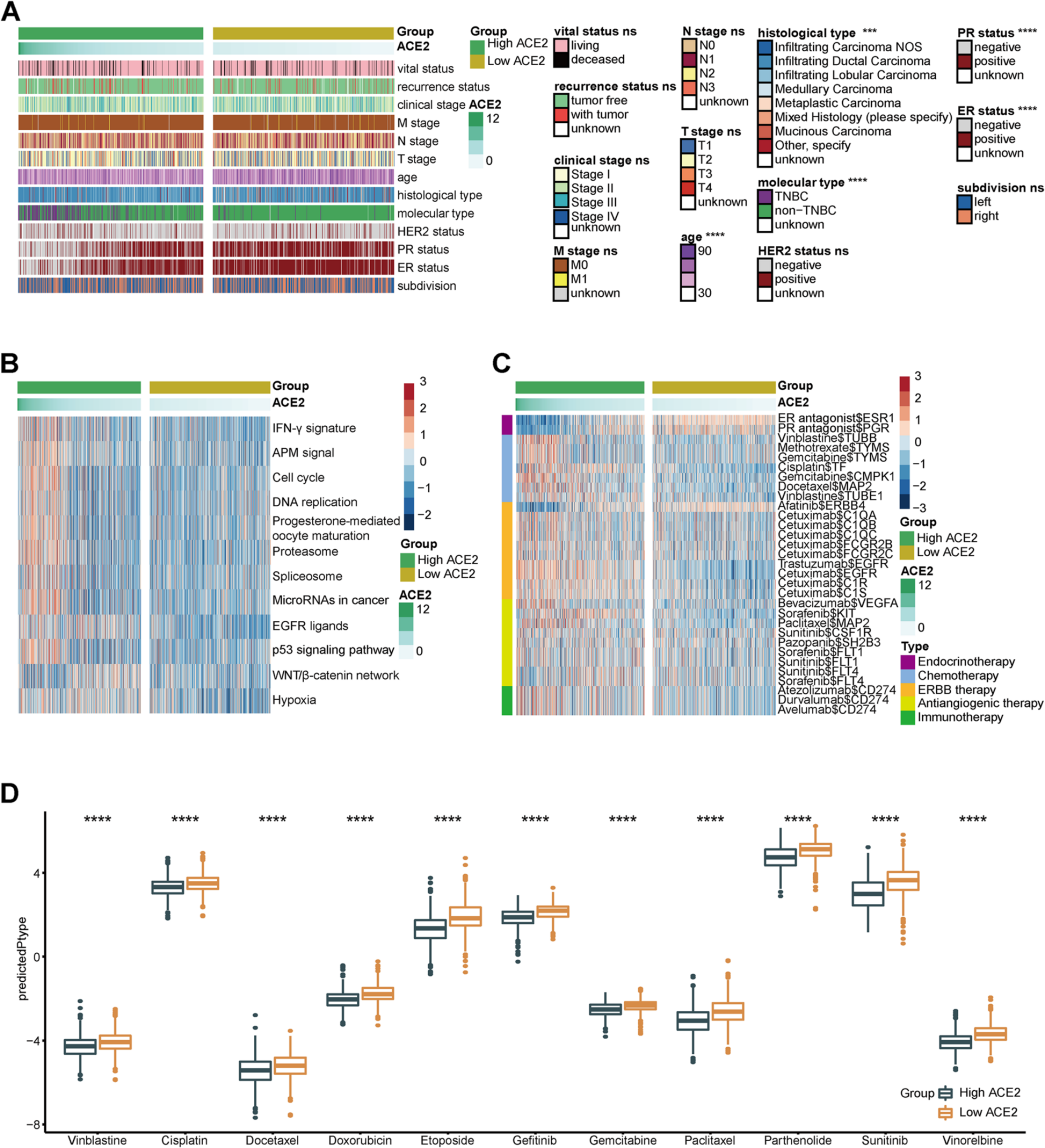
ACE2 predicts the therapeutic options in BC. **A** Correlations between ACE2 and clinic-pathological features in BC. Significance was calculated with Pearson’s Chi-squared test or Student’s t-test. ns-*P* > 0.05, ***P < 0.001, ****P < 0.0001. **B** Correlations between ACE2 and the enrichment scores of several therapeutic signatures. **C** Correlation between ACE2 and the drug-target genes extracted from the Drugbank database. **D** Differences in IC50 of common anti-cancer drugs between the high and low ACE2 groups. Significance was calculated with Student’s t-test. ****P < 0.0001

We next evaluated the ACE2 expression and clinic-pathologic features of BC. As Fig. 3A exhibited, ACE2 expression was significantly associated with age, histological type, molecular type, ER status, and PR status, but not related to other features. In addition, the immunotherapy-related enrichment scores in the high ACE2 group, such as IFN-γ signature, APM signal, cell cycle, and DNA replication, were higher than that in the low ACE2 group (Fig. 3B). Thus, targeted therapy suppressing these oncogenic pathways could be applied for the treatment of BC with high ACE2 expression. Moreover, findings from the Drugbank database revealed a remarkably high response to chemotherapy, anti-ERBB therapy (excluding Afatinib), antiangiogenic therapy and immunotherapy in the high ACE2 group (Fig. 3C). The results showed that chemotherapy, anti-ERBB therapy, antiangiogenic therapy, and immunotherapy could be applied either alone or in combination for the therapy of BC with high ACE2 expression. However, BC with lower ACE2 expression was possibly sensitive to endocrine therapy and Afatinib. Moreover, IC50 of anti-cancer drugs in patients from the TCGA database according to the pRRophetic algorithm was estimated. The results showed patients with high ACE2 expression were liable to sensitive to common anti-cancer drugs (Fig. 3D). To sum up, ACE2 expression identifies TNBC subtype and resistance to endocrine therapy in BC, but patients with high ACE2 expression tend to be sensitive to more therapeutic opportunities, including chemotherapy, anti-ERBB therapy, antiangiogenic therapy, and immunotherapy.

### ACE2 predicts immune phenotypes and clinical subtypes in independent cohorts

The above findings were subsequently validated in the METABRIC database. The expression of most immunomodulators and infiltration levels of the majority of immune cells were increased in the high ACE2 group using various algorithms (Figure S6A-B). Compared with the low ACE2 group, the high ACE2 group had enhanced ESTIMATE Score, Immune Score, and Stromal Score, but decreased Tumor Purity (Figure S6C). ACE2 was positively related to the marker genes of immune cells (Figure S6D). In addition, the positive correlations between ACE2 and the activities of the cancer immunity cycle as well as inhibitory immune checkpoints were also validated in the METABRIC dataset (Figure S6E-F). As expected, immunotargets and T cell inflamed scores were increased in the high ACE2 group (Figure S7A-B). We also analyzed the association between TMB level and ACE2 expression. Although the TMB levels were not remarkably different in the two groups, the notably various mutant feature of TP53 was observed in the METABRIC database (Figure S7C-E). The deficient frequency of MLH1 was high, which implied the MSI-H might be common in the high ACE2 group (Figure S7F). In addition, ACE2 was associated with immunotherapy-related enrichment scores and drug targets expressions based on the evidence from the Drugbank database (Figure S8A-B). Furthermore, we also performed IC50 prediction of anti-cancer drugs in patients from the METABRIC database using the pRRophetic algorithm. As expected, patients with high ACE2 expression were more sensitive to these anti-cancer drugs which were mentioned in the previous analysis (Figure S8C).

**Fig. 4:**
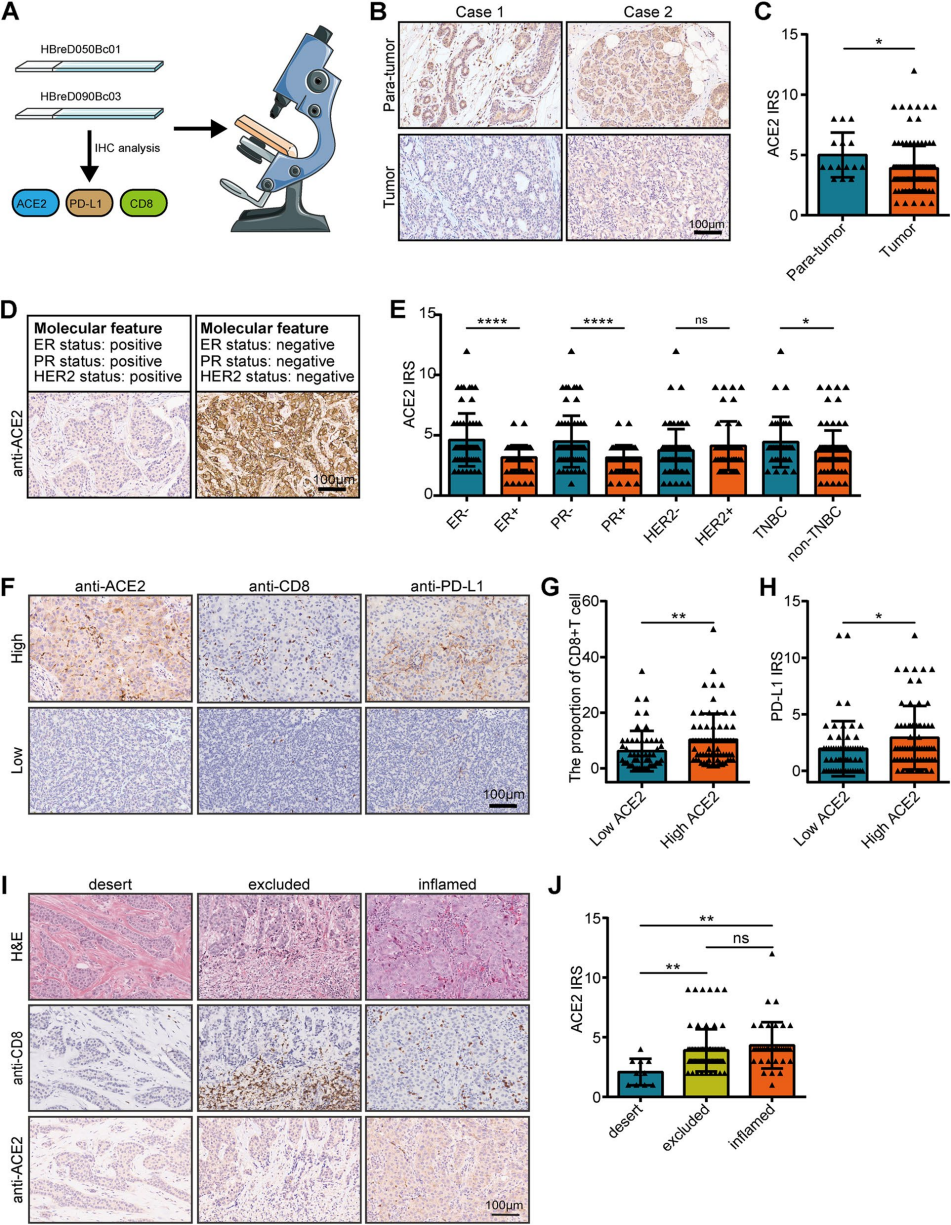
Roles of ACE2 in predicting clinical and immune phenotypes in the recruited TMA cohort. **A** Schematic protocol of validation on the TMA cohort. **B** Representative images revealing ACE2 expression in tumor and para-tumor tissues using anti-ACE2 staining. Magnification, 200 × . **C** Expression levels of ACE2 in tumor and para-tumor tissues. Significance was calculated with Mann Whitney test for ER and PR status and Student’s t-test for HER2 and TNBC status. *P < 0.05. **D** Representative images revealing ACE2 expression in BC tissues with different molecular features. Magnification, 200 × . **E** Expression levels of ACE2 in various molecular subtypes. Significance was calculated with Student’s t-test. *P < 0.05. **F** Representative images revealing CD8^+^ T cell infiltration and PD-L1 expression in the high and low ACE2 groups. Magnification, 200 × . **G** Differences in CD8^+^ T cell infiltration between the high and low ACE2 groups. Significance was calculated with Student’s t-test. *P < 0.05. **H** Differences in PD-L1 expression between the high and low ACE2 groups. Significance was calculated with Student’s t-test. *P < 0.05. **I** Representative images uncovering CD8.^+^ T cell infiltration and ACE2 expressions in BC tissues with different immuno-phenotypes. Magnification, 200 × . **J** Expression of ACE2 in BC with different immuno-phenotypes. Significance was calculated with One-way ANOVA with Tukey’s multiple comparisons test. ns-*P* > 0.05, **P < 0.01, ***P < 0.001

To further validate the above results, we obtained a TMA cohort for verification (Fig. 4A). ACE2 was notably decreased in BC tissues in comparison to normal tissues (Fig. 4B-C). In accord with the above results, ACE2 was overexpressed in ER-negative, PR-negative, and the TNBC tissues (Fig. 4D-E). In addition, the current BC cohort was divided into the low and high expression groups according to the median of ACE2 expression (IRS ≤ 3 vs. IRS > 3). As Table S6 exhibited, ACE2 expression was associated with age, ER status, PR status, and molecular type (Table S6). Moreover, the infiltrating level of CD8^+^ T cell and PD-L1 expression in the high ACE2 group were higher than that in the low ACE2 group (Fig. 4F-H). We also divided the in-house BC patients into the three immuno-phenotypes based on the spatial distribution of CD8^+^ T cells, and ACE2 expression was the lowest in the desert tumors (F ig. 4I-J). In conclusion, ACE2 expression is related to clinical features and immune phenotypes in BC.

### Ang-1–7 sensitizes the response to chemotherapy and immunotherapy by remodeling the TME

**Fig. 5:**
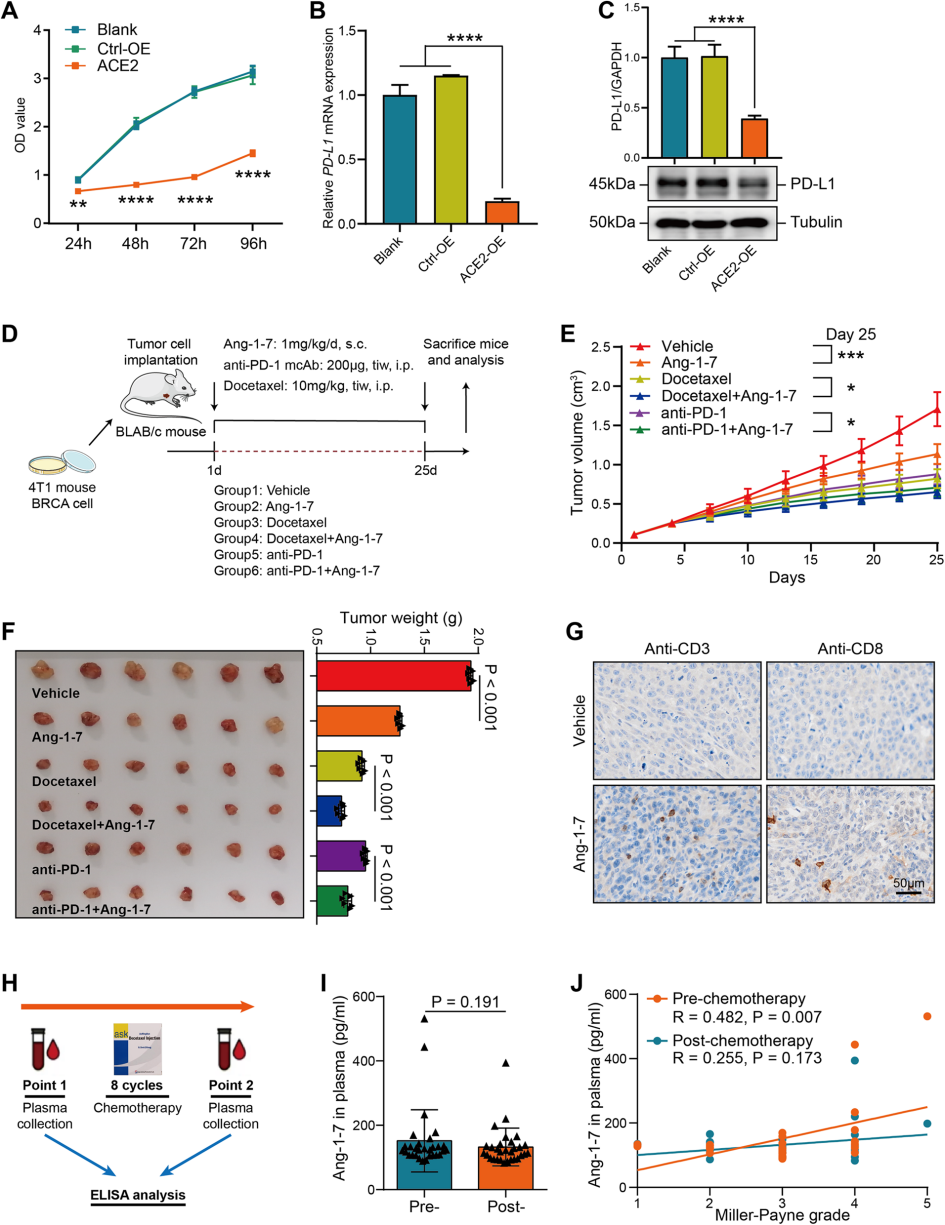
Ang-1–7 sensitizes the response to chemotherapy and immunotherapy. **A** Proliferative potential of MDA-MB-231 cells was checked using CCK-8 assay after ACE2 overexpression. **B** The transcriptional level of PD-L1 was checked using qPCR after ACE2 overexpression. **C** The protein level of PD-L1 was checked using Western blotting after ACE2 overexpression. **D** Schematic protocol of the combinated therapy in immunocompetent BLAB/c mice. **E** Tumor volume was monitored every three days. Significance was calculated with One-way ANOVA with Tukey’s multiple comparisons test. *P < 0.05, ***P < 0.001. **F** The tumor weight of isolated tumors from these six groups, mice were sacrificed on day 25 after treatment. Significance was calculated with One-way ANOVA with Tukey’s multiple comparisons test. ***P < 0.001, ****P < 0.0001. **G** Relative infiltration level of CD3^+^ and CD8^+^ T cells in control and Ang-1–7 treated tumors. Magnification, 200 × . **H** Schematic diagram of time point of plasma sample collection. **I** Comparison of plasma Ang-1–7 levels pre-and post-chemotherapy. Significance was calculated with Student’s t-test. ns-*P* > 0.05. **J** Correlation between the response to neoadjuvant chemotherapy and plasma Ang-1–7 levels pre-and post-chemotherapy. Significance was calculated with Pearson correlation analysis

We next evaluated the effect of ACE2 on PD-L1 expression and the proliferation of MDA-MB-231 cells. First, the efficiency of ACE2 overexpression was validated by qPCR and Western blotting (Figure S9A-B). ACE2 overexpression significantly inhibited cell proliferation and downregulated PD-L1 expression in BRCA cells (Fig. 5A-C). Ang-1–7 has been reported to mediate ACE2-induced molecular functions [5, 7]. Thus, we evaluated the pharmacodynamic effects of Ang-1–7 on tumor growth, chemotherapy, and immunotherapy (Fig. 5D). In the mouse model, Ang-1–7 sensitized mouse transplanted BRCA to the chemotherapy and anti-PD-1 immunotherapy, that revealed its significant anti-tumor effect (Fig. 5E-F). We speculated that Ang-1–7 remodeled the TME by turning “cold” to “hot” tumors. IHC analysis uncovered that Ang-1–7 significantly increased the infiltrating abundance of CD3^+^ and CD8^+^ immune cells (Fig. 5G). We also compared the expression of several effective (IFN-γ, CXCL9, CXCL10) and suppressive (TGF-β1, IL-10) cytokines in the control and Ang-1–7 treated tumors in mouse models. The results showed that Ang-1–7 upregulated CXCL9 and downregulated TGF-β1, but did not affect other cytokines expression (Figure S10A-E). Thus, we speculated that Ang-1–7 promoted the infiltration of effective immune cells and inhibited the infiltration of inhibitory immune cells by regulating the expression and secretion of multiple cytokines. Moreover, we collected 30 plasma samples from BC patients treated with neoadjuvant chemotherapy (Fig. 5H). Neoadjuvant chemotherapy did not change the levels of Ang-1–7 (Fig. 5I). Moreover, a high plasma level of Ang-1–7 pre-chemotherapy was associated with a good response to neoadjuvant chemotherapy in the cohort (Fig. 5J). Overall, Ang-1–7 exerts an anti-tumor role in BC and sensitizes the response to chemotherapy and immunotherapy by turning “cold” to “hot” tumors.

## Discussion

ACE2 is a tumor suppressor in various cancers via regulating Ang-(1–7)/MasR axis, involved in the proliferation, invasion and migration, tumor-associated angiogenesis, and EMT processes [35]. It has been reported that ACE2 highly expresses in epithelial cells in colorectal tumors express, indicating its significant role in cancers [36]. Dai et al*.* [8] reports that the upregulated ACE2 expression is correlated with a favorable clinical outcome in hepatocellular carcinoma. The associations between ACE2 with anti-tumor immunity and immunotherapy were also explored. Yang et al*.* [9] reports that the overexpression of ACE2 is significantly associated with enhanced immune infiltration in endometrial cancer and renal papillary cell cancer. Bao et al*.* [29] reveals a tight correlation between ACE2 expression and immune gene signatures in multiple cancers. However, the values of ACE2 in the identification of tumor immune status have not been evaluated.

In this research, we first conducted a pan-cancer analysis of the immunological features of ACE2 and discovered that ACE2 exhibited the closest correlations with immunological factors in BC. We found that ACE2 was positively related to the expression of important immunomodulators. ACE2 was also positively related to increased TIICs and cancer immunity cycles. The recruitment of effector TIICs was enhanced, thereby facilitated the formation of an inflamed TME. We discovered that high ACE2 expression was correlated with the enhanced response to immunotherapy by checking the difference of immune checkpoints expressions, T cell inflamed score, TMB, MMR protein deficiency status and IPS scores in the high and low ACE2 groups. Another important finding was that high ACE2 levels indicated the negativity of hormone receptors, including ER, PR, and HER2 receptors. More importantly, ACE2 expression was increased in the TNBC subtype of BC. TNBC is summarized by deadly aggressiveness and lacked treatment [37]. However, overexpressed PD-L1 and high levels of TIICs are commonly observed in TNBC [38], and its response to immune checkpoint inhibition is encouraging. In addition, the expression of ACE2 is regulated by inflammatory cytokines [39, 40]. We speculate that this may be a reason why ACE2 is upregulated in inflamed tumors, especially in TNBC. Moreover, TNBC expressed higher PD-L1, which is the suitable subtype for the study of anti-tumor immunity in BC [41, 42]. Thus, the role of ACE2 in regulating anti-tumor immunity in TNBC should be given particular attention.

Previous researches have confirmed the anti-inflammatory role of ACE2 via its enzymatic product Ang-1–7 [43, 44]. Anti-inflammatory drugs such as COX2 inhibitors exhibits exciting effects on synergistically enhancing the efficacy of immunotherapy [45]. In this research, we found ACE2 overexpression inhibited PD-L1 expression, suggesting ACE2/Ang-1–7 axis might be helpful for effector immune cells infiltration. The pharmacodynamic effects of Ang-1–7 on tumor growth, chemotherapy and immunotherapy were evaluated in BALB/c mouse BC models. Consistent with previous research, Ang-1–7 exhibited a tumor-suppressive effect. Ang-1–7 also sensitized Docetaxel chemotherapy and anti-PD-1 immunotherapy. However, we observed that the anti-tumor effect of Ang-1–7 was not as good as that of Docetaxel. Therefore, Ang-1–7 might be an adjuvant drug for chemotherapy and immunotherapy, rather than an independent anti-tumor drug. Moreover, IHC analysis uncovered that Ang-1–7 significantly increased the infiltrating abundance of CD3^+^ and CD8^+^ immune cells. Increased immune cells infiltration adaptively upregulated PD-L1 expressed on tumor cells, which could account for the inconsistencies between the results of bioinformatics and IHC analysis that ACE2 was correlated with PD-L1 expression and the findings of in vitro assays that ACE2 inhibited PD-L1 expression.

However, the current research also has several unresolved doubts. First, although we found that ACE2 was immunologically associated, these results were not justifying that ACE2 shaped an inflamed TME in BC as mechanistic evidence was not explored. Besides, bioinformatics evidence suggested that high ACE2 expression was associated with nearly all recruited different immune cell types including suppressive ones (Tregs, MDSCs). To further validate the role of Ang-1–7 in regulating the recruitment of immune cells, we compared the expression of several effective (IFN-γ, CXCL9, CXCL10) and suppressive (TGF-β1, IL-10) cytokines in the control and Ang-1–7 treated groups. The results showed that Ang-1–7 upregulated CXCL9 and downregulated TGF-β1. Thus, we speculated that Ang-1–7 promoted the infiltration of effector immune cells and inhibited the infiltration of inhibitory immune cells. Moreover, although the results uncovered that ACE2 showed the highest correlations with immunomodulators, TIICs, and immune checkpoints in BC, whether Ang-1–7 could modulate the TME in other cancers was still unknown and needed to be further explored.

## Conclusions

Overall, we reveal that ACE2 is associated with an inflamed TME according to the evidence of the positive relationship between ACE2 and the immunological patterns of TME in BC. In addition, ACE2 is notably associated with molecular subtypes of BC and the response to several therapeutic strategies. Moreover, Ang-1–7 exerts an anti-tumor effect by remodeling the TME and sensitizes BC to the chemotherapy and immunotherapy. ACE2 may be a promising biomarker for the identification of immunological features in BC, and its enzymatic product Ang-1–7 could be used as a potential anti-tumor drug.

## Supplementary Information

**Additional file 1:** **Figure S1.** Pan-cancer analysis of ACE2 expression in tumor and para-tumor tissues in the TCGA database. Significance was calculated with Student’s t-test. ns-*P*>0.05, *P<0.05, **P<0.01, ***P<0.001, ****P < 0.0001. **Figure S2. **Pan-cancer analysis of the correlations between ACE2 and immunological features. (A) Correlations between ACE2 and 122 immunomodulators (chemokines, receptors, MHC and immunostimulators). The color reveals the correlation coefficient. The asterisks reveal statistical differences assessed by Pearson analysis. (B) Correlations between ACE2 and 28 TIICs calculated with the ssGSEA algorithm. The color reveals the correlation coefficient. The asterisks reveal statistical differences assessed by Pearson analysis. (C) Correlations between ACE2 and four immune checkpoints, LAG3, TIGIT, CTLA4, PD-L1. The dots symbolize cancer types. **Figure S3. **Expression patterns of ACE2 in different cell types in BC tissues. (A) Expression levels of ACE2 in different cell types in BC tissues in GSE143423 and SRP114962 datasets. (B) Representative images revealing ACE2 expression in BC tissues using anti-ACE2 staining. Magnification, 200×.** Figure S4. **Potential regulatory factors of ACE2 in BC.(A) Mutations in ACE2 gene. (B) The associations between CNV pattern and ACE2 expression in BC. Significance was calculated with One-way ANOVA. (C) The correlation between methylation level and ACE2 expression. Significance was calculated with Pearson’s correlation analysis. **Figure S5. **Mutational density curve in BC.The TMB levels were most enriched in the range of 0-1200. **Figure S6. **ACE2 predicts an inflamed TME in BC, the validated results in the METABRIC dataset. (A) Expression levels of 122 immunomodulators between the high and low ACE2 groups in BC. (B) Differences in the levels of TIICs calculated using five algorithms between the high and low ACE2 groups. (C) Differences in Tumor Purity, ESTIMATE Score, Immune Score, andStromal Score estimating by ESTIMATE method between the high and low ACE2 groups. Significance was calculated with Student’s t-test. ns-*P>*0.05, ***P<0.001, ****P < 0.0001. (D) Differences in the gene markers of the common TIICs between the high and low ACE2 groups. (E) Differences in the various steps of the cancer immunity cycle between the high and low ACE2 groups. Significance was calculated with Student’s t-test. ns-*P>*0.05, *P<0.05, **P<0.01, ***P<0.001, ****P < 0.0001. (F) Correlation between ACE2 and common inhibitory immune checkpoints. Significance was calculated with Pearson correlation analysis. The color reveals the Pearsoncorrelation coefficient. **Figure S7. **Correlations between ACE2 and the immune phenotype in BC, the validated results in the METABRIC dataset. (A) Differences in expression levels of immune-related targets the high and lowACE2 groups in BC. Significance was calculated with Student’s t-test. *P<0.05, ****P < 0.0001. (B) Differences in T cell inflamed scores between the high and low ACE2 groups. The T cell inflamed score is positively related to the response to cancer immunotherapy. Significance was calculated with Student’s t-test. ****P < 0.0001. (C, D) Mutational landscape in the high and low ACE2 groups. (E) Differences in TMB levelsbetween the high and low ACE2 groups. Significance was calculated with Student’s t-test. (F) Differences in deficiency rates of MMR proteins between the high and low ACE2 groups. Significance was calculated with Pearson’s Chi-squared test. **Figure S8. **ACE2 predicts the therapeutic options in BC, the validated results in the METABRIC dataset. (A) Expression level of ACE2in different molecular subtypes in BC. (B) Diagnostic values of ACE2 expression in identifying ER, PR and triple-negative subtypes. (C) Correlations between ACE2 and the enrichment scores of several therapeutic signatures. (D) Correlation between ACE2 and the drug-target genes extracted from the Drugbank database. (E) Differences in IC50 of common anti-cancer drugs between the high and low ACE2 groups. Significance was calculated with Student’s t-test. **P < 0.0001, ****P < 0.0001. **Figure S9. **Validation of the efficiency of ACE2 overexpression. (A) The efficiency of ACE2 overexpression was checked by qPCR. (B) The efficiency of ACE2 overexpression was checked by western blotting. Significancewas calculated with Student’s t-test. **P < 0.01, ****P < 0.0001. **Figure S10. **Expression of several cytokines in control and Ang-1-7 treated tumors obtainedfrom the mouse models.(A) IFN-γ, (B) CXCL9, (C) CXCL10, (D) TGF-β1, (E) IL-10. Significance was calculated with Mann Whitney test for A, B, C, and E and Student’s t-test for D. ns-*P>*0.05, *P < 0.05.

**Additional file 2: Table S1. **Table of abbreviations in the TCGA database. **Table S2. **Summary of sample size of public datasets. **Table S3. **Detailed information of immunotherapy-related gene signatures. **Table S4. **Detailed information of BC patients receiving Paclitaxel-dependent neoadjuvant chemotherapy. **Table S5. **GO and KEGG pathway enrichment analyses of ACE2 in BC. **Table S6. **Association between ACE2 expression and clinic-pathological features in BC. 

## Data Availability

All data supported the results in this study are showed in this published article and its supplementary files. Besides, original data for bioinformatics analysis could be downloaded from corresponding platforms.

## Abbreviations

ACE2: Angiotensin-converting enzyme 2
RNA-seq: RNA sequencing
Ang-1–7: Angiotensin-(1–7)
BC: Breast cancer
TIICs: Tumor-infiltrating immune cells
TME: Tumor microenvironment
TCGA: The Cancer Genome Atlas
CNV: Copy number variant
TF: Transcription factor
TMB: Tumor mutation burden
MHC: Major histocompatibility complex
GO: Gene Ontology
KEGG: Kyoto Encyclopedia of Genes and Genomes
GSEA: Gene set enrichment analysis
ssGSEA: Single sample gene set enrichment analysis
IPS: Immunophenoscore
TCIA: The Cancer Immunome Atlas
CGP: Cancer Genome Project
IC50: Half-maximal inhibitory concentration
GDSC: Genomics of Drug Sensitivity in Cancer
TMA: Tissue microarray
IHC: Immunohistochemistry
IRS: Immunoreactivity score
ELISA: Enzyme-linked immunosorbent assay
qRT-PCR: Quantitative real‑time PCR
CCK-8: Cell Counting Kit-8
ROC: Receiver-operating characteristic
AUC: The area under the ROC curve
OS: Overall survival
PFS: Progression-free survival
MSI-H: Microsatellite instability
MMR: Mismatch repair
TNBC: Triple-negative breast cancer
