# Supplementary material for: Angiotensin-converting enzyme 2 identifies immuno-hot tumors suggesting angiotensin-(1–7) as a sensitizer for chemotherapy and immunotherapy in breast cancer
Source: Biol Proced Online. 2022 Oct 25;24:15. doi: 10.1186/s12575-022-00177-9 (PMC9594906; doi:10.1186/s12575-022-00177-9)
Supplement: Supplementary file 1 — Additional file 1: Figure S1. Pan-cancer analysis of ACE2 expression in tumor and para-tumor tissues in the TCGA database. Significance was calculated with Student’s t-test. ns-P>0.05, *P<0.05, **P<0.01, ***P<0.001, ****P < 0.0001. Figure S2. Pan-cancer analysis of the correlations between ACE2 and immunological features. (A) Correlations between ACE2 and 122 immunomodulators (chemokines, receptors, MHC and immunostimulators). The color reveals the correlation coefficient. The asterisks reveal statistical differences assessed by Pearson analysis. (B) Correlations between ACE2 and 28 TIICs calculated with the ssGSEA algorithm. The color reveals the correlation coefficient. The asterisks reveal statistical differences assessed by Pearson analysis. (C) Correlations between ACE2 and four immune checkpoints, LAG3, TIGIT, CTLA4, PD-L1. The dots symbolize cancer types. Figure S3. Expression patterns of ACE2 in different cell types in BC tissues. (A) Expression levels of ACE2 in different cell types in BC tissues in GSE143423 and SRP114962 datasets. (B) Representative images revealing ACE2 expression in BC tissues using anti-ACE2 staining. Magnification, 200×. Figure S4. Potential regulatory factors of ACE2 in BC.(A) Mutations in ACE2 gene. (B) The associations between CNV pattern and ACE2 expression in BC. Significance was calculated with One-way ANOVA. (C) The correlation between methylation level and ACE2 expression. Significance was calculated with Pearson’s correlation analysis. Figure S5. Mutational density curve in BC.The TMB levels were most enriched in the range of 0-1200. Figure S6. ACE2 predicts an inflamed TME in BC, the validated results in the METABRIC dataset. (A) Expression levels of 122 immunomodulators between the high and low ACE2 groups in BC. (B) Differences in the levels of TIICs calculated using five algorithms between the high and low ACE2 groups. (C) Differences in Tumor Purity, ESTIMATE Score, Immune Score, andStromal Score estimating by ESTIMATE m [file 12575_2022_177_MOESM1_ESM.docx]

**Supplemental Figures**


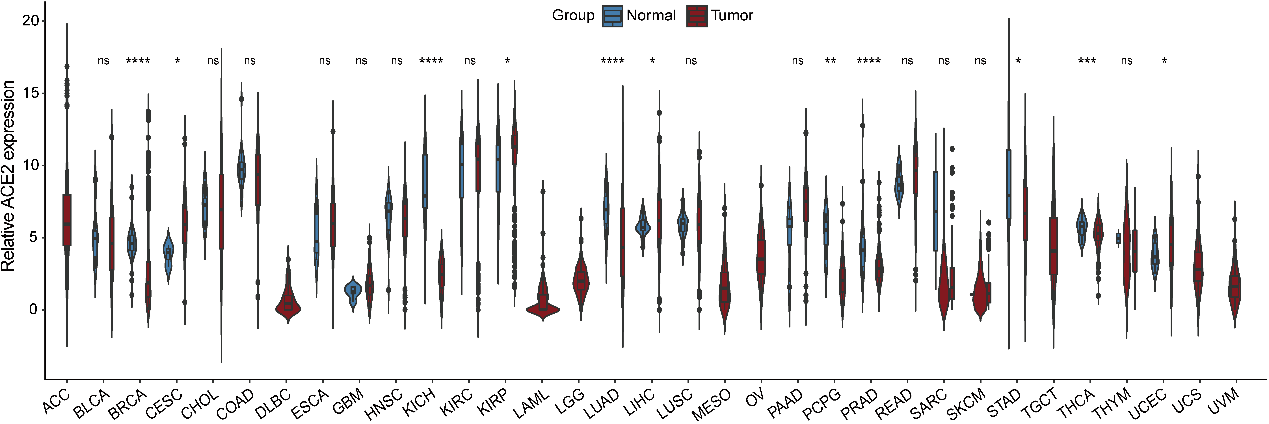


**Figure S1. Pan-cancer analysis of ACE2 expression in tumor and para-tumor tissues in the TCGA database.** Significance was calculated with Student’s t-test. ns-P>0.05, *P<0.05, **P<0.01, ***P<0.001, ****P < 0.0001.


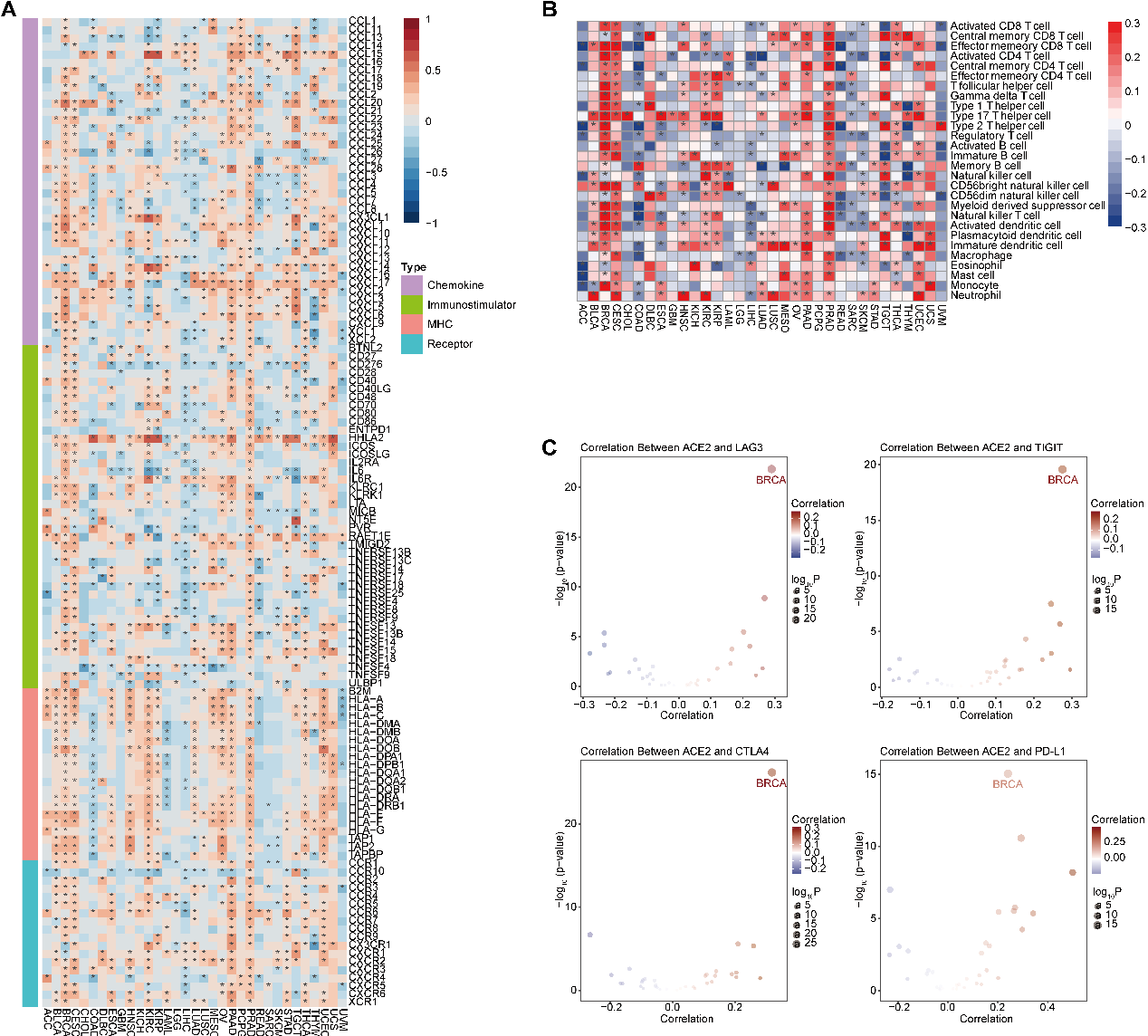


**Figure S2. Pan-cancer analysis of the correlations between ACE2 and immunological features.**

(A) Correlations between ACE2 and 122 immunomodulators (chemokines, receptors, MHC and immunostimulators). The color reveals the correlation coefficient. The asterisks reveal statistical differences assessed by Pearson analysis. (B) Correlations between ACE2 and 28 TIICs calculated with the ssGSEA algorithm. The color reveals the correlation coefficient. The asterisks reveal statistical differences assessed by Pearson analysis. (C) Correlations between ACE2 and four immune checkpoints, LAG3, TIGIT, CTLA4, PD-L1. The dots symbolize cancer types.


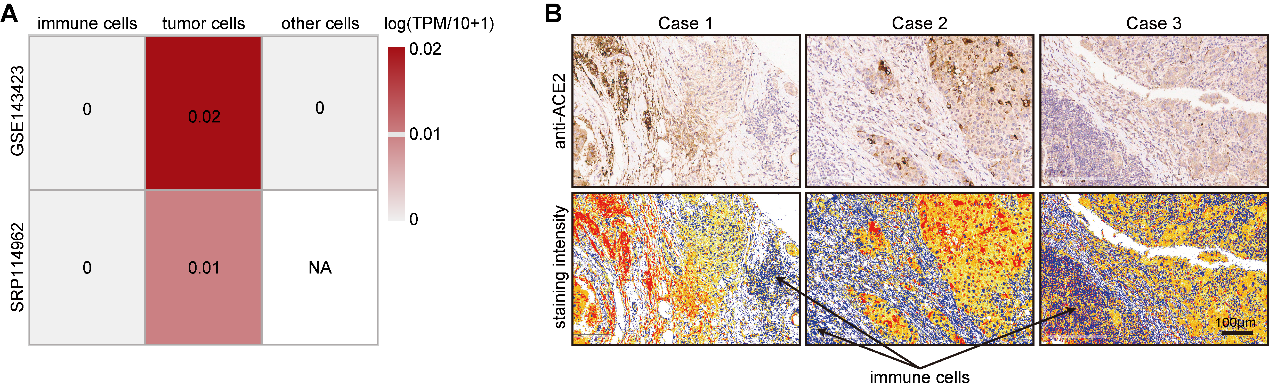


**Figure S3. Expression patterns of ACE2 in different cell types in BC tissues.**

(A) Expression levels of ACE2 in different cell types in BC tissues in GSE143423 and SRP114962 datasets. (B) Representative images revealing ACE2 expression in BC tissues using anti-ACE2 staining. Magnification, 200×.


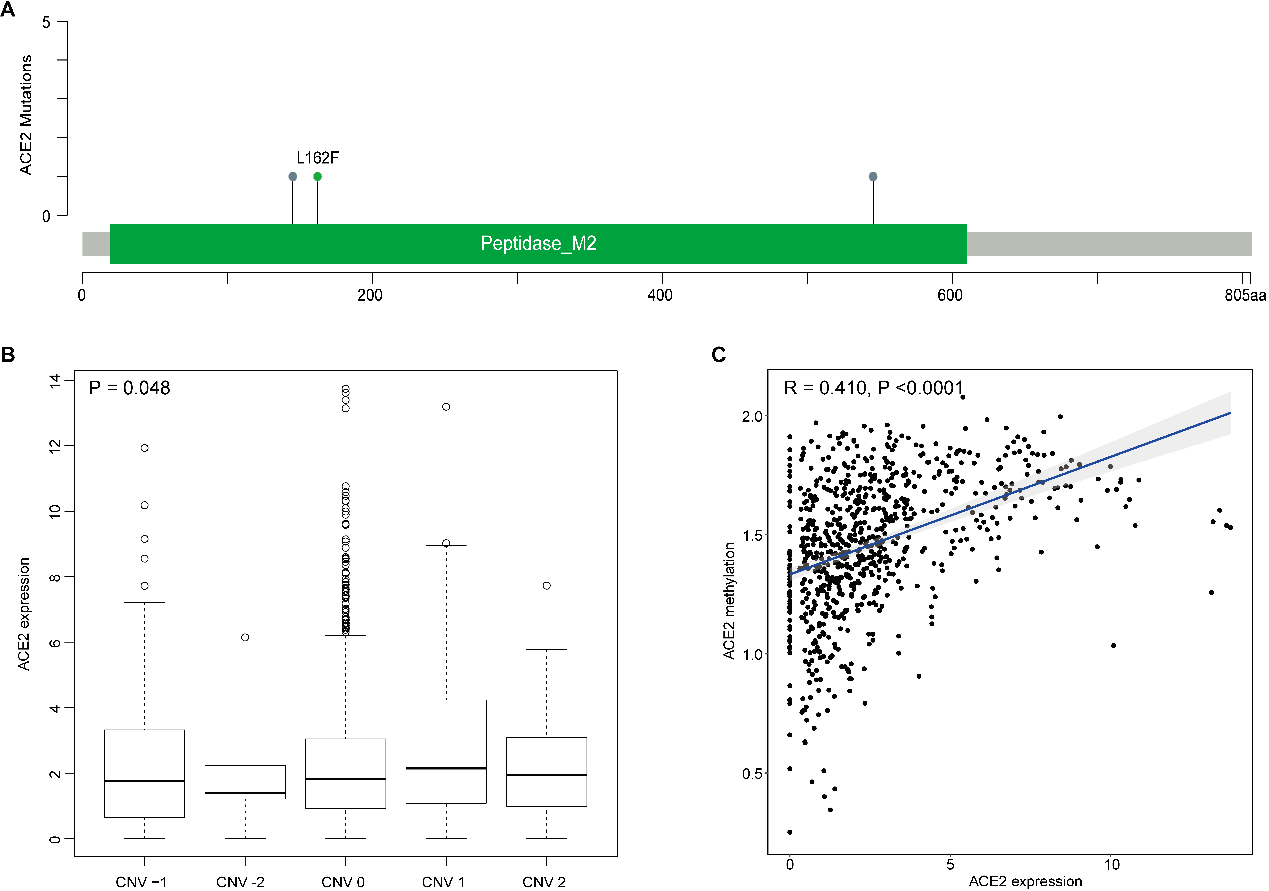


**Figure S4. Potential regulatory factors of ACE2 in BC.**

(A) Mutations in ACE2 gene. (B) The associations between CNV pattern and ACE2 expression in BC. Significance was calculated with One-way ANOVA. (C) The correlation between methylation level and ACE2 expression. Significance was calculated with Pearson’s correlation analysis.

**
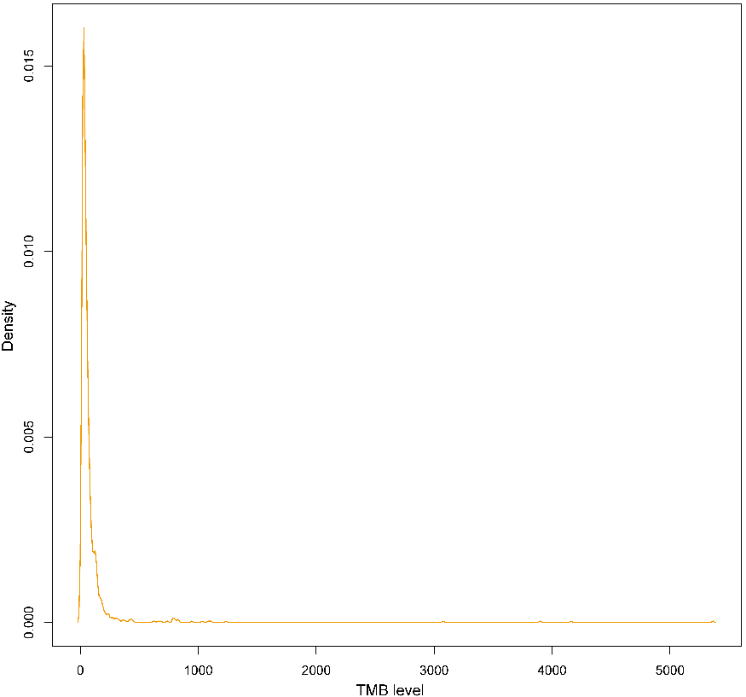
**

**Figure S5. Mutational density curve in BC.**

The TMB levels were most enriched in the range of 0-1200.


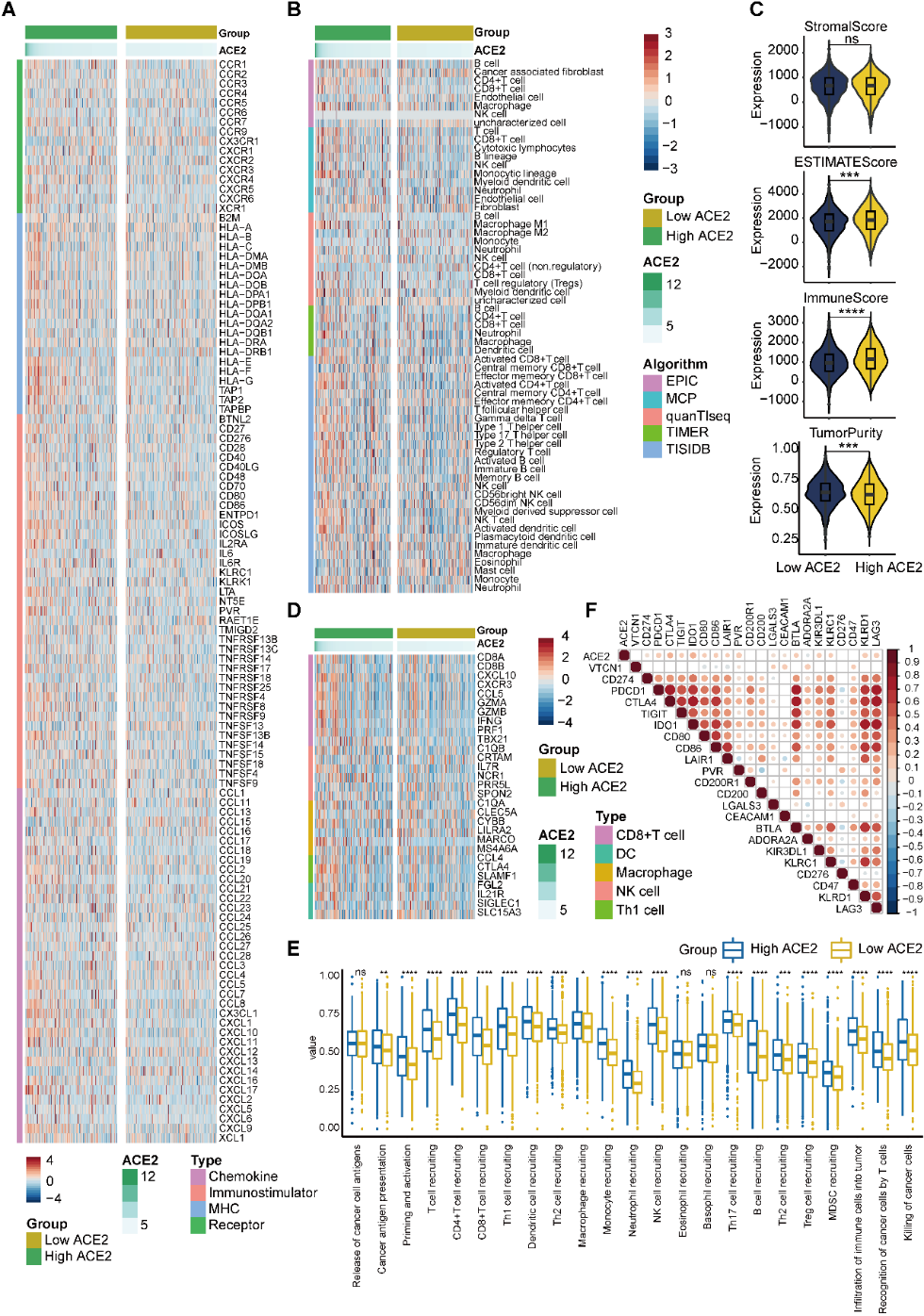


**Figure S6. ACE2 predicts an inflamed TME in BC, the validated results in the METABRIC dataset.**

(A) Expression levels of 122 immunomodulators between the high and low ACE2 groups in BC. (B) Differences in the levels of TIICs calculated using five algorithms between the high and low ACE2 groups. (C) Differences in Tumor Purity, ESTIMATE Score, Immune Score, and Stromal Score estimating by ESTIMATE method between the high and low ACE2 groups. Significance was calculated with Student’s t-test. ns-P>0.05, ***P<0.001, ****P < 0.0001. (D) Differences in the gene markers of the common TIICs between the high and low ACE2 groups. (E) Differences in the various steps of the cancer immunity cycle between the high and low ACE2 groups. Significance was calculated with Student’s t-test. ns-P>0.05, *P<0.05, **P<0.01, ***P<0.001, ****P < 0.0001. (F) Correlation between ACE2 and common inhibitory immune checkpoints. Significance was calculated with Pearson correlation analysis. The color reveals the Pearson correlation coefficient.


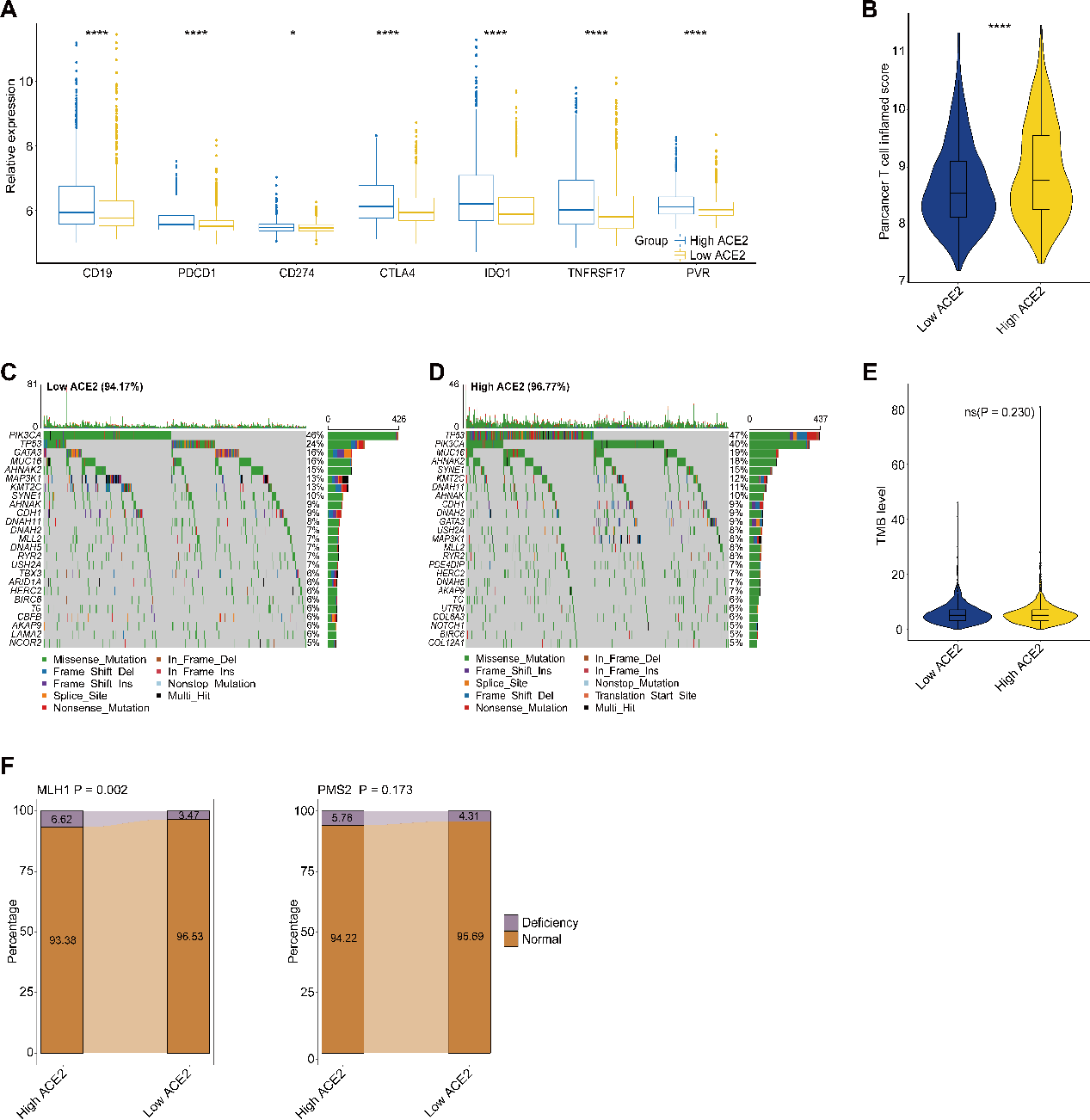


**Figure S7. Correlations between ACE2 and the immune phenotype in BC, the validated results in the METABRIC dataset.**

(A) Differences in expression levels of immune-related targets the high and low ACE2 groups in BC. Significance was calculated with Student’s t-test. *P<0.05, ****P < 0.0001. (B) Differences in T cell inflamed scores between the high and low ACE2 groups. The T cell inflamed score is positively related to the response to cancer immunotherapy. Significance was calculated with Student’s t-test. ****P < 0.0001. (C, D) Mutational landscape in the high and low ACE2 groups. (E) Differences in TMB levels between the high and low ACE2 groups. Significance was calculated with Student’s t-test. (F) Differences in deficiency rates of MMR proteins between the high and low ACE2 groups. Significance was calculated with Pearson’s Chi-squared test.


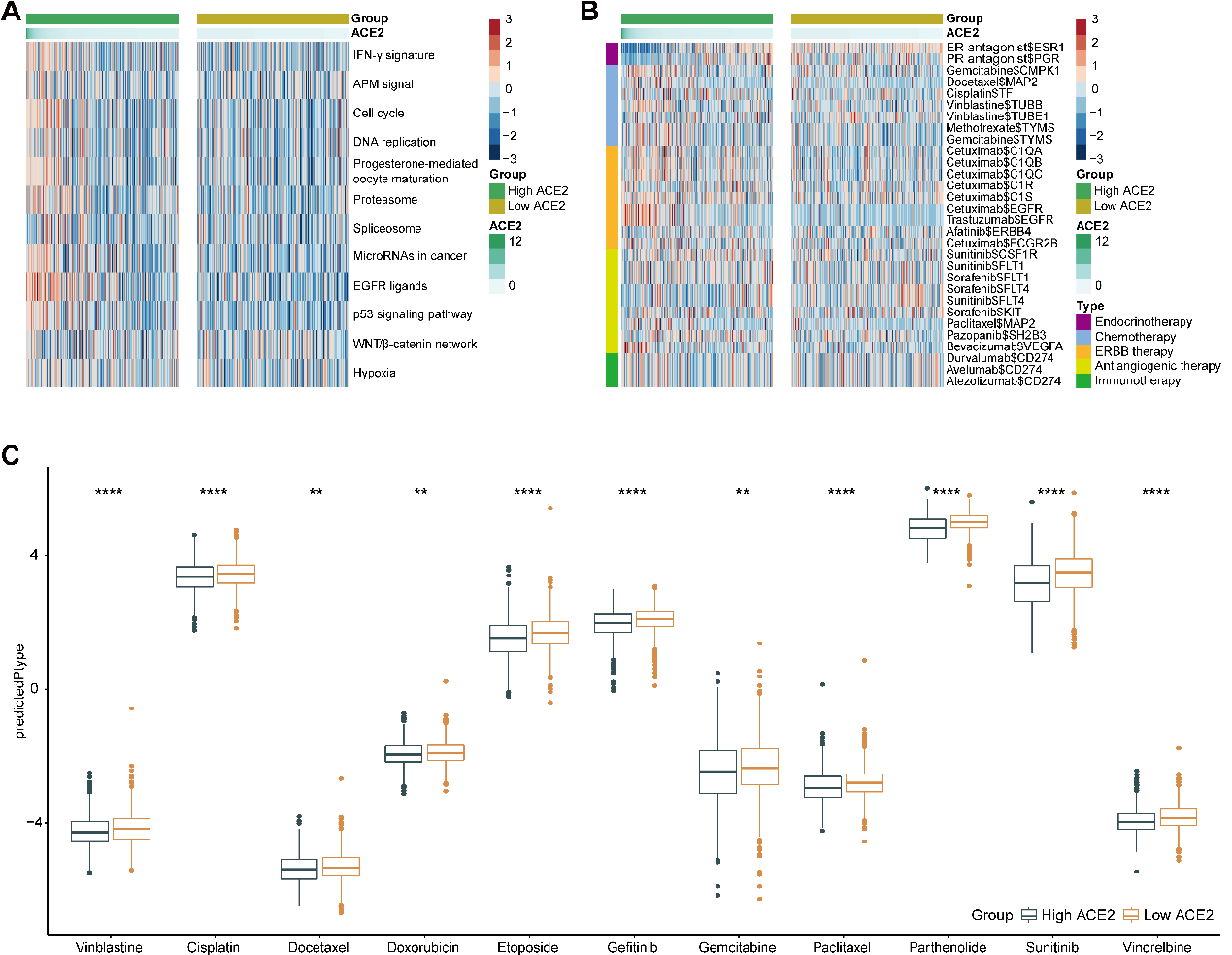


**Figure S8. ACE2 predicts the therapeutic options in BC, the validated results in the METABRIC dataset.**

(A) Expression level of ACE2 in different molecular subtypes in BC. (B) Diagnostic values of ACE2 expression in identifying ER, PR and triple-negative subtypes. (C) Correlations between ACE2 and the enrichment scores of several therapeutic signatures. (D) Correlation between ACE2 and the drug-target genes extracted from the Drugbank database. (E) Differences in IC50 of common anti-cancer drugs between the high and low ACE2 groups. Significance was calculated with Student’s t-test. **P < 0.0001, ****P < 0.0001.


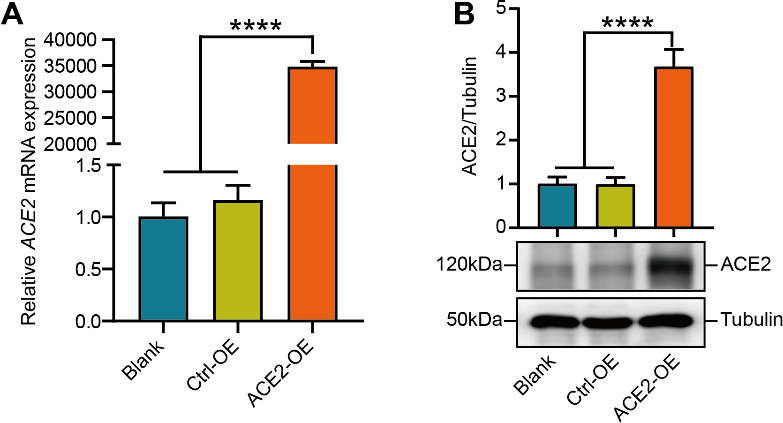


**Figure S9. Validation of the efficiency of ACE2 overexpression.**

(A) The efficiency of ACE2 overexpression was checked by qPCR. (B) The efficiency of ACE2 overexpression was checked by western blotting. Significance was calculated with Student’s t-test. **P < 0.01, ****P < 0.0001.


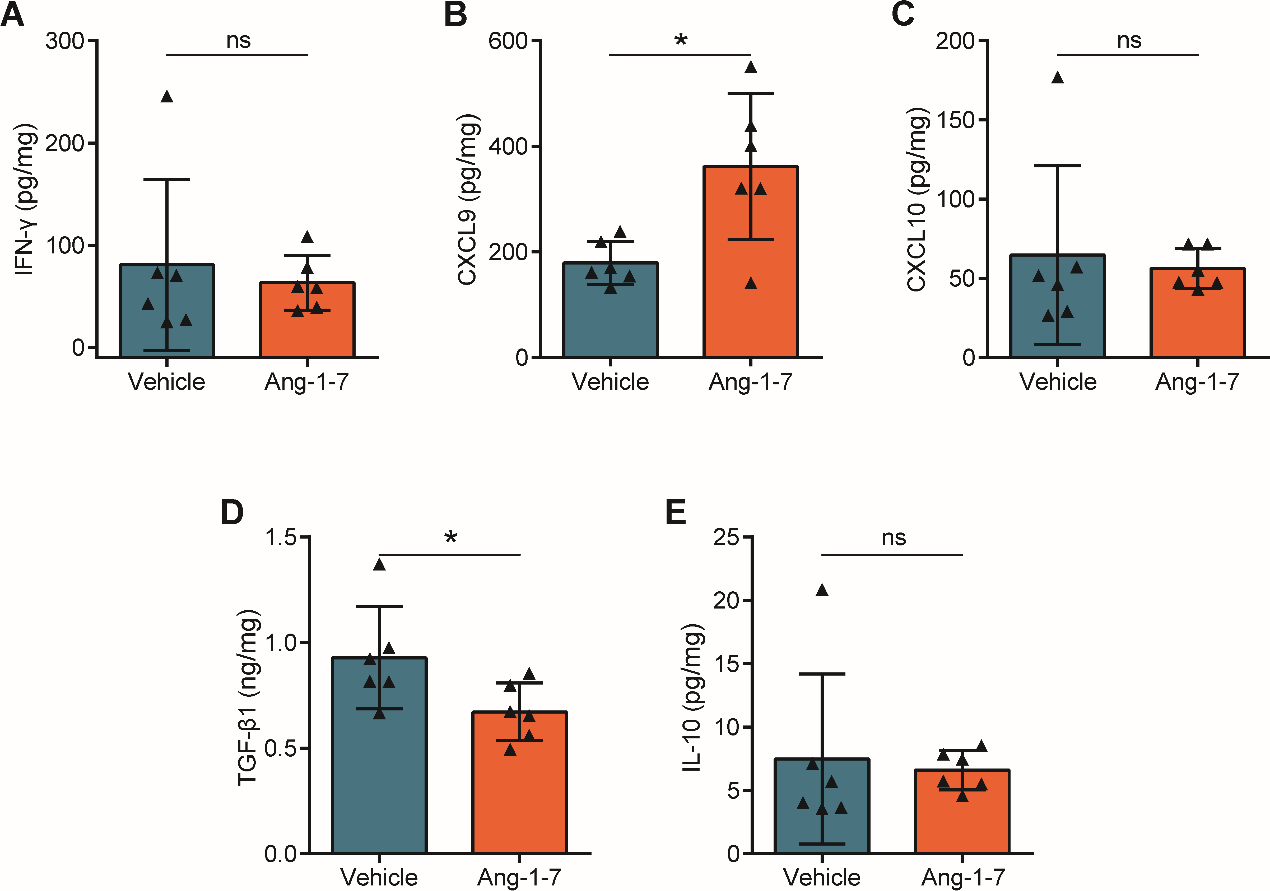


**Figure S10. Expression of several cytokines in control and Ang-1-7 treated tumors obtained from the mouse models.**

(A) IFN-γ, (B) CXCL9, (C) CXCL10, (D) TGF-β1, (E) IL-10. Significance was calculated with Mann Whitney test for A, B, C, and E and Student’s t-test for D. ns-P>0.05, *P < 0.05.
