# Supplementary material for: Angiotensin-converting enzyme 2 identifies immuno-hot tumors suggesting angiotensin-(1–7) as a sensitizer for chemotherapy and immunotherapy in breast cancer
Source: Biol Proced Online. 2022 Oct 25;24:15. doi: 10.1186/s12575-022-00177-9 (PMC9594906; doi:10.1186/s12575-022-00177-9)
Supplement: Supplementary file 2 — Additional file 2: Table S1. Table of abbreviations in the TCGA database. Table S2. Summary of sample size of public datasets. Table S3. Detailed information of immunotherapy-related gene signatures. Table S4. Detailed information of BC patients receiving Paclitaxel-dependent neoadjuvant chemotherapy. Table S5. GO and KEGG pathway enrichment analyses of ACE2 in BC. Table S6. Association between ACE2 expression and clinic-pathological features in BC. [file 12575_2022_177_MOESM2_ESM.docx]

**Supplementary Tables**

**Table S1. Table of abbreviations in the TCGA database.**

| Abbreviation | Full name |
| --- | --- |
| ACC | Adrenocortical carcinoma |
| BLCA | Bladder urothelial carcinoma |
| BRCA | Breast invasive carcinoma |
| CESC | Cervical squamous cell carcinoma and endocervical adenocarcinoma |
| CHOL | Cholangio carcinoma |
| COAD | Colon adenocarcinoma |
| DLBC | Lymphoid neoplasm diffuse large B-cell lymphoma |
| ESCA | Esophageal carcinoma |
| GBM | Glioblastoma multiforme |
| HNSC | Head and neck squamous cell carcinoma |
| KICH | Kidney chromophobe carcinoma |
| KIRC | Kidney renal clear cell carcinoma |
| KIRP | Kidney renal papillary cell carcinoma |
| LAML | Acute myeloid leukemia |
| LGG | Brain lower grade glioma |
| LIHC | Liver hepatocellular carcinoma |
| LUAD | Lung adenocarcinoma |
| LUSC | Lung squamous cell carcinoma |
| MESO | Mesothelioma |
| OV | Ovarian serous cystadenocarcinoma |
| PAAD | Pancreatic adenocarcinoma |
| PCPG | Pheochromocytoma and paraganglioma |
| PRAD | Prostate adenocarcinoma |
| READ | Rectum adenocarcinoma |
| SARC | Sarcoma |
| SKCM | Skin cutaneous melanoma |
| STAD | Stomach adenocarcinoma |
| TGCT | Testicular germ cell tumors |
| THCA | Thyroid carcinoma |
| THYM | Thymoma |
| UCEC | Uterine corpus endometrial carcinoma |
| UCS | Uterine carcinosarcoma |
| UVM | Uveal melanoma |

**Table S2. Summary of sample size of public datasets.**

| Dataset | Tumor sample | Normal sample |
| --- | --- | --- |
| TCGA-ACC | 79 | 0 |
| TCGA-BLCA | 407 | 19 |
| TCGA-BRCA | 1090 | 114 |
| TCGA-CESC | 305 | 3 |
| TCGA-CHOL | 36 | 9 |
| TCGA-COAD | 288 | 41 |
| TCGA-DLBC | 48 | 0 |
| TCGA-ESCA | 185 | 11 |
| TCGA-GBM | 167 | 5 |
| TCGA-HNSC | 522 | 44 |
| TCGA-KICH | 66 | 25 |
| TCGA-KIRC | 534 | 72 |
| TCGA-KIRP | 291 | 32 |
| TCGA-LAML | 173 | 0 |
| TCGA-LGG | 530 | 0 |
| TCGA-LIHC | 373 | 50 |
| TCGA-LUAD | 517 | 59 |
| TCGA-LUNG | 1019 | 110 |
| TCGA-LUSC | 502 | 51 |
| TCGA-MESO | 87 | 0 |
| TCGA-OV | 308 | 0 |
| TCGA-PAAD | 179 | 4 |
| TCGA-PCPG | 184 | 3 |
| TCGA-PRAD | 498 | 52 |
| TCGA-READ | 95 | 10 |
| TCGA-SARC | 263 | 2 |
| TCGA-SKCM | 473 | 1 |
| TCGA-STAD | 415 | 35 |
| TCGA-TGCT | 156 | 0 |
| TCGA-THCA | 513 | 59 |
| TCGA-THYM | 120 | 2 |
| TCGA-UCEC | 177 | 24 |
| TCGA-UCS | 57 | 0 |
| TCGA-UVM | 80 | 0 |
| METABRIC cohort | 1904 | 0 |

**Table S3. Detailed information of immunotherapy-related gene signatures.**

| Pathway | Reference | Gene markers |
| --- | --- | --- |
| IFN-γ signature | PMID: 28650338 | TIGIT, CD27, CD8A, PDCD1LG2, LAG3, CD274, CXCR6, CMKLR1, NKG7, CCL5, PSMB10, IDO1, CXCL9, HLA-DQA1, CD276, STAT1, HLA-DRB1, HLA-E |
| APM signal | PMID: 31563503 | B2M, HLA-A, HLA-B, HLA-C, TAP1, TAP2 |
| Cell cycle | PMID: 29443960 | BUB1, BUB1B, CCNA2, CCNB2, CCNE1, CCNE2, CDC20, CDC25A, CDC25C, CDC6, CDK1, CDK2, CDKN2A, DBF4, E2F1, E2F2, ESPL1, MAD2L1, MAD2L2, MCM2, MCM4, MCM6, MCM7, ORC1, ORC6, PCNA, PLK1, SKP2, SMC3, TFDP1, TTK, YWHAB |
| DNA replication | PMID: 29443960 | DNA2, FEN1, LIG1, MCM2, MCM4, MCM6, MCM7, PCNA, POLA2, POLE, POLE2, PRIM1, PRIM2, RFC2, RFC3, RFC4, RFC5, RNASEH2A, RPA1, RPA3 |
| Progesterone-mediated oocyte maturation | PMID: 29443960 | BUB1, CCNA2, CCNB2, CDC25A, CDC25C, CDK1, CDK2, MAD2L1, MAD2L2, PLK1 |
| Proteasome | PMID: 29443960 | IFNG, PSMA4, PSMB2, PSMB4, PSMC4, PSMD4, PSMD7 |
| Spliceosome | PMID: 29443960 | HNRNPM, LSM3, LSM4, LSM5, MAGOHB, PRPF19, SF3B2, SF3B3, SF3B4, SNRNP40, SNRPA1, SNRPC, USP39, WBP11 |
| MicroRNAs in cancer | PMID: 29443960 | BRCA1, CCNE1, CCNE2, CDC25A, CDC25C, CDCA5, CDKN2A, DNMT1, E2F1, E2F2, EZH2, KIF23, STMN1, TRIM71 |
| EGFR ligands | PMID: 31563503 | EGFR, AREG, AREGB, EREG, HBEGF, TGFA |
| p53 signaling pathway | PMID: 29443960 | CCNB2, CCNE1, CCNE2, CDK1, CDK2, CDKN2A, GTSE1, PPM1D, RFWD2, RRM2 |
| WNT/β-catenin network | PMID: 27197067 | CTNNB1, TFF1, HAPLN1, IHH, WNT7B, BMP7, SEMA5A, SCN5A, ERBB3, TSPAN8, EPCAM, TH, GPX2, GAD1, HSD17B2, KRT7, NOX1, CYB5A, CYP4F12, ID4, SIM2, MECOM, MSX2, KLF5, SMAD6, POU5F1, FOXQ1, GATA2, GATA3, EMX2 |
| Hypoxia | PMID: 31563503 | CAV1, COL5A1, ITGA5, P4HA2, SLC16A1, TGFBI, DPYSL2, SRPX, TRAM2, SYDE1, LRP1, PDLIM2, SAV1, AHNAK2, CAD, CYP1B1, DAAM1, DSC2, SLC2A3, FUT11, GLG1, GULP1, LDLR, THBS4 |

**Table S4. Detailed information of BC patients receiving Paclitaxel-dependent neoadjuvant chemotherapy.**

| ID | Age | Molecular subtype | ER status | PR status | HER2 status | Miller-Payne grade | T stage | N stage | M stage | Clinical stage | Chemotherapy regimens |
| --- | --- | --- | --- | --- | --- | --- | --- | --- | --- | --- | --- |
| Sample1 | 42 | Luminal B | + | + | + | 4 | T1 | N0 | M0 | 1 | EC-TH*8/21 |
| Sample2 | 38 | TNBC | - | - | - | 3 | T1 | N0 | M0 | 1 | EC-T*8/14 |
| Sample3 | 55 | HER2 | - | - | + | 4 | T1 | N0 | M0 | 1 | EC*4/14-TH*4/21 |
| Sample4 | 62 | Luminal B | + | - | + | 4 | T1 | N0 | M0 | 1 | EC*4/14-TH*4/21 |
| Sample5 | 51 | HER2 | - | - | + | 4 | T1 | N0 | M0 | 1 | EC*4/14-TH*4/21 |
| Sample6 | 48 | HER2 | - | - | + | 4 | T1 | N0 | M0 | 1 | EC*4/14-TH*4/21 |
| Sample7 | 49 | Luminal B | + | + | + | 3 | T1 | N0 | M0 | 1 | EC*4/14-TH*4/21 |
| Sample8 | 46 | Luminal A | + | + | - | 5 | T0 | N0 | M0 | 1 | EC-T*8/21 |
| Sample9 | 45 | Luminal B | + | - | + | 3 | T2 | N1 | M0 | 2 | EC-T*8/14 |
| Sample10 | 48 | Luminal A | + | + | - | 1 | T2 | N1 | M0 | 2 | EC-T*8/14 |
| Sample11 | 57 | HER2 | - | - | + | 3 | T1 | N1 | M0 | 2 | TCbHP*8/21 |
| Sample12 | 66 | TNBC | - | - | - | 3 | T1 | N1 | M0 | 2 | EC-T*8/14 |
| Sample13 | 47 | HER2 | - | - | + | 4 | T1 | N1 | M0 | 2 | EC*4/14-TH*4/21 |
| Sample14 | 51 | HER2 | - | - | + | 4 | T1 | N1 | M0 | 2 | EC-TH*8/14 |
| Sample15 | 56 | Luminal A | + | - | - | 2 | T2 | N1 | M0 | 2 | EC-T*8/21 |
| Sample16 | 55 | Luminal A | + | - | - | 3 | T2 | N1 | M0 | 2 | EC-T*8/21 |
| Sample17 | 49 | Luminal A | + | + | - | 3 | T1 | N1 | M0 | 2 | EC-T*8/14 |
| Sample18 | 61 | Luminal B | + | - | + | 4 | T1 | N1 | M0 | 2 | EC-TH*8/14 |
| Sample19 | 50 | Luminal A | + | + | - | 2 | T1 | N2 | M0 | 2 | EC-T*8/21 |
| Sample20 | 51 | HER2 | - | - | + | 2 | T2 | N2 | M0 | 3 | EC*4/14-TH*4/21 |
| Sample21 | 54 | HER2 | - | - | + | 3 | T2 | N3 | M0 | 3 | TCbHP*6/21 |
| Sample22 | 37 | Luminal B | + | + | + | 3 | T2 | N3 | M0 | 3 | EC-TH*8/14 |
| Sample23 | 50 | Luminal A | + | + | - | 2 | T4 | N2 | M0 | 3 | EC-T*8/21 |
| Sample24 | 52 | Luminal A | + | + | - | 2 | T1 | N3 | M0 | 3 | EC-T*8/14 |
| Sample25 | 61 | Luminal A | + | + | - | 3 | T3 | N3 | M0 | 3 | EC-T*8/21 |
| Sample26 | 55 | Luminal A | + | + | - | 3 | T1 | N3 | M0 | 3 | EC-T*8/14 |
| Sample27 | 66 | Luminal A | + | + | - | 3 | T1 | N3 | M0 | 3 | EC-T*8/14 |
| Sample28 | 69 | Luminal A | + | + | - | 2 | T3 | N2 | M0 | 3 | EC-T*8/21 |
| Sample29 | 45 | TNBC | - | - | - | 3 | T2 | N3 | M0 | 3 | EC-T*8/21 |
| Sample30 | 50 | Luminal A | + | + | - | 1 | T1 | N3 | M0 | 3 | EC-T*8/21 |

**Table S5. GO and KEGG pathway enrichment analyses of ACE2 in BC.**

| Gene Set | Description | ES | NES | P value |
| --- | --- | --- | --- | --- |
| Biological process | | | | |
| GO:0002250 | adaptive immune response | 0.592 | 1.974 | <0.001 |
| GO:0002237 | response to molecule of bacterial origin | 0.576 | 1.900 | <0.001 |
| GO:1990868 | response to chemokine | 0.655 | 1.895 | <0.001 |
| GO:0060326 | cell chemotaxis | 0.568 | 1.867 | <0.001 |
| GO:0031349 | positive regulation of defense response | 0.559 | 1.865 | <0.001 |
| Cell component | | | | |
| GO:0005790 | smooth endoplasmic reticulum | 0.717 | 1.749 | <0.001 |
| GO:0001533 | cornified envelope | 0.689 | 1.898 | <0.001 |
| GO:0097038 | perinuclear endoplasmic reticulum | 0.682 | 1.544 | 0.020 |
| GO:0001891 | phagocytic cup | 0.671 | 1.533 | 0.019 |
| GO:0042611 | MHC protein complex | 0.666 | 1.452 | 0.023 |
| Molecular function | | | | |
| GO:0016701 | oxidoreductase activity, acting on single donors with incorporation of molecular oxygen | 0.774 | 1.779 | <0.001 |
| GO:0016917 | GABA receptor activity | 0.755 | 1.670 | <0.001 |
| GO:0042287 | MHC protein binding | 0.725 | 1.707 | <0.001 |
| GO:0001530 | lipopolysaccharide binding | 0.720 | 1.689 | 0.003 |
| GO:0070003 | threonine-type peptidase activity | 0.688 | 1.563 | 0.024 |
| KEGG | | | | |
| hsa04650 | Natural killer cell mediated cytotoxicity | 0.654 | 1.987 | <0.001 |
| hsa04060 | Cytokine-cytokine receptor interaction | 0.599 | 1.969 | <0.001 |
| hsa05340 | Primary immunodeficiency | 0.741 | 1.889 | <0.001 |
| hsa00601 | Glycosphingolipid biosynthesis | 0.803 | 1.875 | <0.001 |
| hsa00380 | Tryptophan metabolism | 0.726 | 1.843 | <0.001 |

ES: Enrichment score; NES: Normalized enrichment score.

**Table S6. Association between ACE2 expression and clinic-pathological features in BC.**

| Features | Cases | ACE2 expression | | χ^2^ value | P value |
| --- | --- | --- | --- | --- | --- |
|  |  | low | high |  |  |
| Age |  |  |  |  |  |
| ≤60 | 94 | 48 | 46 | 4.561 | 0.033 |
| >60 | 31 | 9 | 22 |  |  |
| T stage |  |  |  |  |  |
| ≤2cm | 47 | 18 | 29 | 1.979 | 0.159 |
| >2cm | 76 | 39 | 37 |  |  |
| unknown | 2^a^ |  |  |  |  |
| N stage |  |  |  |  |  |
| N0 | 63 | 27 | 36 | 0.275 | 0.600 |
| N1-N3 | 61 | 29 | 32 |  |  |
| unknown | 1^b^ |  |  |  |  |
| M stage |  |  |  |  |  |
| N0 | 124 | 56 | 68 | 1.203 | 0.456^c^ |
| M1 | 1 | 1 | 0 |  |  |
| Clinical stage |  |  |  |  |  |
| 0-2 | 89 | 41 | 48 | 0.027 | 0.869 |
| 3-4 | 36 | 16 | 20 |  |  |
| Grade |  |  |  |  |  |
| I-II | 53 | 28 | 25 | 1.755 | 0.185 |
| II-III | 71 | 29 | 42 |  |  |
| unknown | 1 |  |  |  |  |
| ER status |  |  |  |  |  |
| negative | 63 | 18 | 45 | 14.847 | <0.001 |
| positive | 62 | 39 | 23 |  |  |
| PR status |  |  |  |  |  |
| negative | 70 | 21 | 49 | 15.607 | <0.001 |
| positive | 55 | 36 | 19 |  |  |
| HER2 status |  |  |  |  |  |
| negative | 78 | 35 | 43 | 0.102 | 0.750 |
| positive | 46 | 22 | 24 |  |  |
| unknown | 1 |  |  |  |  |
| Molecular type |  |  |  |  |  |
| non-TNBC | 88 | 47 | 41 | 6.758 | 0.009 |
| TNBC | 36 | 10 | 26 |  |  |
| unknown | 1 |  |  |  |  |

Note: a: T1-T3, not T4; b: The patient had distant metastasis; c: P value was calculated by Fisher test.
